# A dual target molecular magnetic resonance imaging probe for noninvasive profiling of pathologic alpha-synuclein and microgliosis in a mouse model of Parkinson’s disease

**DOI:** 10.3389/fnins.2024.1428736

**Published:** 2024-07-24

**Authors:** Xianwei Sun, Andrew Badachhape, Prajwal Bhandari, Jeannie Chin, Ananth Annapragada, Eric Tanifum

**Affiliations:** ^1^Department of Radiology, Baylor College of Medicine, Houston, TX, United States; ^2^Department of Neuroscience, Baylor College of Medicine, Houston, TX, United States; ^3^Department of Radiology, Texas Children’s Hospital, Houston, TX, United States

**Keywords:** imaging of alpha-synuclein, MRI of neuroinflammation, MRI of alpha-synuclein, molecular MRI of microgliosis, accelerated alpha-synuclein clearance

## Abstract

The pathogenesis of Parkinson’s disease (PD) is characterized by progressive deposition of alpha-synuclein (α-syn) aggregates in dopaminergic neurons and neuroinflammation. Noninvasive *in vivo* imaging of α-syn aggregate accumulation and neuroinflammation can elicit the underlying mechanisms involved in disease progression and facilitate the development of effective treatment as well as disease diagnosis and prognosis. Here we present a novel approach to simultaneously profile α-syn aggregation and reactive microgliosis *in vivo*, by targeting oligomeric α-syn in cerebrospinal fluid with nanoparticle bearing a magnetic resonance imaging (MRI), contrast payload. In this proof-of-concept report we demonstrate, *in vitro*, that microglia and neuroblastoma cell lines internalize agglomerates formed by cross-linking the nanoparticles with oligomeric α-syn. Delayed *in vivo* MRI scans following intravenous administration of the nanoparticles in the M83 α-syn transgenic mouse line show statistically significant MR signal enhancement in test mice versus controls. The *in vivo* data were validated by *ex-vivo* immunohistochemical analysis which show strong correlation between *in vivo* MRI signal enhancement, Lewy pathology distribution, and microglia activity in the treated brain tissue. Furthermore, neuronal and microglial cells in brain tissue from treated mice display strong cytosolic signal originating from the nanoparticles, attributed to *in vivo* cell uptake of nanoparticle/oligomeric α-syn agglomerates.

## Graphical abstract

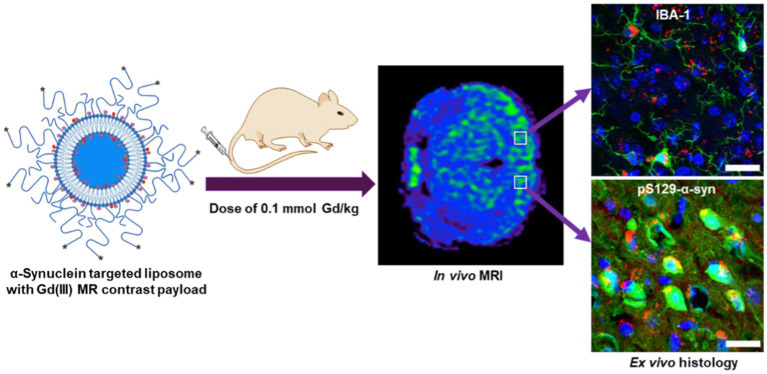

## 1 Introduction

Parkinson’s disease (PD) is the leading movement disorder and the second most common neurodegenerative disorder. Current statistics also show that it is the fastest growing neurological disease worldwide, with a 95% increase in incidence from 1990–2019, a growth rate that surpasses that of Alzheimer’s disease (AD) (Ou et al., 2021). In the United States alone, an estimated 1.5 million individuals suffer from the disease, with 50 to 60 thousand new cases reported each year (Willis et al., 2022). Globally, an estimated 6.2 million individuals suffer from PD, a number projected to double by 2040 (Ou et al., 2021). Despite this pressing need, the pathophysiological mechanisms underlying disease initiation and progression that can lead to the development of disease-modifying therapies remain elusive (Dorsey et al., 2018; Ou et al., 2021; Willis et al., 2022). In fact, some experts now believe that PD should be classified as a pandemic to heighten activism, focused planning, resource mobilization and novel approaches for the detection, treatment, and management of the disease (Dorsey and Bloem, 2018).

The pathogenesis of PD mirrors that of related neurological disorders, which are characterized by pathological deposits of misfolded protein aggregates, neuronal death, chronic neuroinflammation, and compromise of the blood-brain barrier (BBB) (Dugger and Dickson, 2017). In PD and other movement disorders, generally referred to as synucleinopathies (such as dementia with Lewy bodies and multiple system atrophy), the pathological protein aggregates observed in postmortem brain tissue sections appear as cytosolic inclusions in neurons and glia (referred to as Lewy bodies and Lewy neurites) (Koga et al., 2021). Studies on regional distribution in postmortem PD brain tissue suggest that Lewy pathology originates in the olfactory system, peripheral autonomic nervous system, and dorsal motor nucleus of the glossopharyngeal and vagal nerves and progressively spreads to other areas of the central nervous system (CNS). In addition, the presence of Lewy pathology in the medulla oblongata, pontine tegmentum, and anterior olfactory bulb predates the clinical manifestations of PD-related motor symptoms. These symptoms first appear when the pathology has spread to the substantia nigra and other foci within the basal portions of the mid- and forebrain (Braak et al., 2003; Beach et al., 2009; Adler et al., 2019), suggesting that technologies that can help in the detection and quantification of the pathologic variants of α-syn and reactive gliosis can have significant impact on early detection, prognosis, as well as the development of disease modifying treatments.

The dominant protein component in Lewy pathology consists of α-synuclein (α-syn) aggregates. Under normal physiological conditions, α-syn adopts a helical conformation, but under pathological conditions, it misfolds and aggregates into β-sheets (Bendor et al., 2013). The prevailing theory is that disease propagation proceeds through cell-to-cell transmission of α-syn aggregates mediated by reactive gliosis (Dexter and Jenner, 2013; Zhang et al., 2018; Wakabayashi, 2020; Mavroeidi and Xilouri, 2021). In normal individuals, α-syn is expressed predominantly in neurons, but in the diseased brain, activated glia may internalize pathological aggregates via receptor-mediated and phagocytotic pathways (Mavroeidi and Xilouri, 2021; Chavarría et al., 2022). Neuronal cells release small amounts of monomeric and oligomeric α-syn by exocytosis (Mavroeidi and Xilouri, 2021), and oligomeric species have been shown to trigger reactive gliosis (Ingelsson, 2016). Levels of total α-syn in the cerebrospinal fluid (CSF) and blood may not vary significantly between normal individuals and PD patients, but there is strong evidence that the fraction of oligomeric aggregates increases with disease severity and plays a significant role in disease pathogenesis (Lee et al., 2014; Ingelsson, 2016; Parnetti et al., 2019; Chavarría et al., 2022). Genetically engineered mouse models of this disease, such as the A53T α-synuclein transgenic line M83, develop an age-dependent regional distribution of intracytoplasmic neuronal α-syn inclusions that parallel disease onset and progression in humans (Giasson et al., 2002; Lee et al., 2002). Although these models do not perfectly match the exact human condition, they serve as excellent tools for the drug discovery process.

While there has been considerable success in the development of protocols to detect and quantify oligomeric α-syn in CSF and blood samples with high accuracy (Parnetti et al., 2019), noninvasive *in vivo* molecular imaging tools for reactive gliosis and pathologic α-syn in the brain, which can be more impactful in the development of disease-modifying therapies, remain elusive. To date, most research effort on noninvasive imaging of α-syn aggregates has been focused on small organic molecule-based positron emission tomography (PET) or single-photon emission computed tomography (SPECT) tracers for intracellular Lewy pathology (Korat et al., 2021; Alzghool et al., 2022; Roshanbin et al., 2022). However, none of these have been successful in clinical trials compared to PET tracers for the extracellular amyloid-β (Aβ) plaques in AD, which have recently been approved for clinical use by the US Food and Drug Administration (FDA) (Pemberton et al., 2022). Several PET tracers targeting the translocator protein (TSPO) which is upregulated in reactive microglia are also in development as a tool for imaging neuroinflammation in PD and other neurodegenerative disorders but have all faced different challenges in clinical translation (Corica et al., 2023).

**Figure 1:**
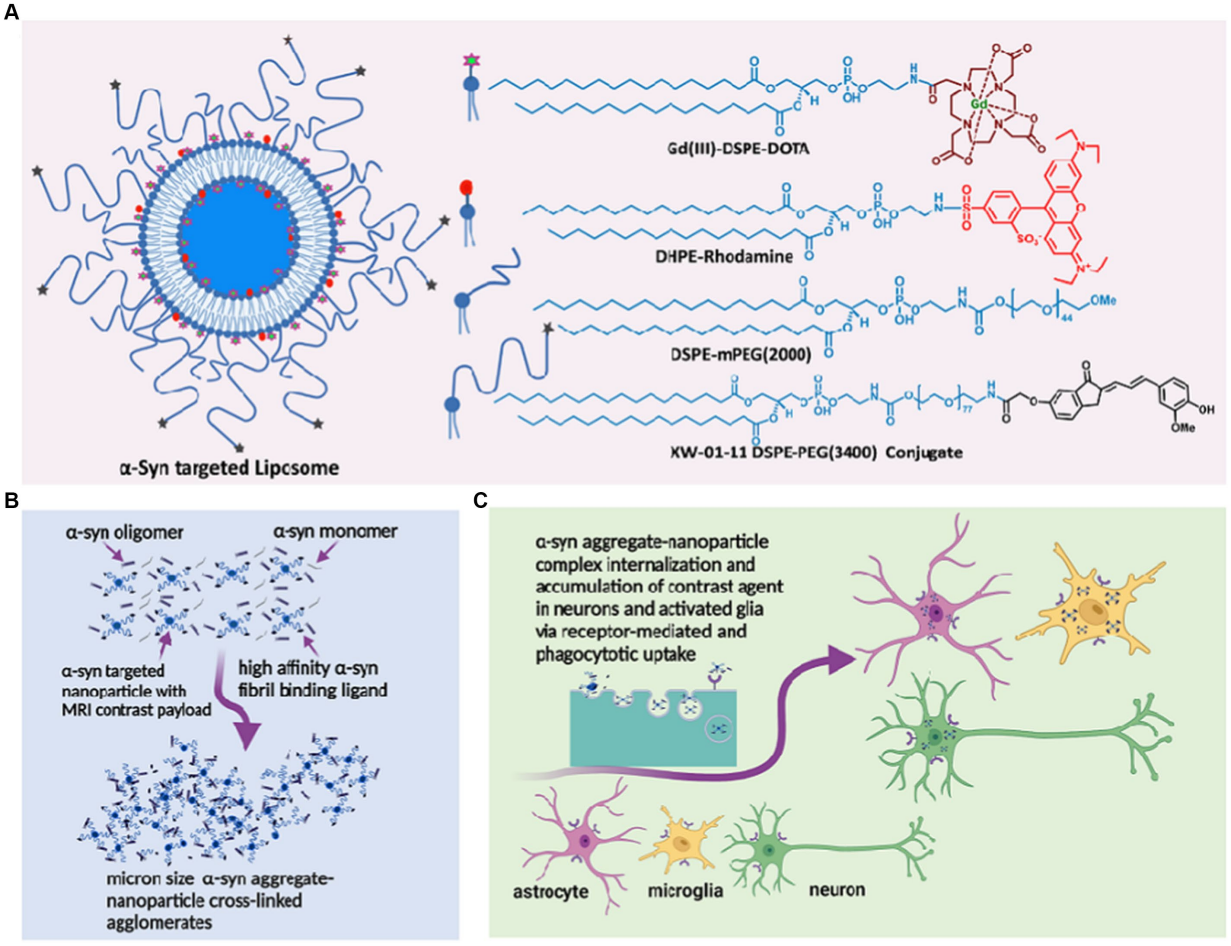
α-synuclein nano scavenger hypothesis. **(A)** Liposome nanoparticles labeled with oligomeric α-synbinding ligands (nano scavenger) and *in vivo* and *ex vivo* imaging contrast payloads. **(B)** Nano scavengers cross-link oligomeric α-syn to form NS/oligomeric α-syn agglomerates in the CSF micro environment. **(C)** Activated glia and neurons internalize agglomerates by conventional endocytosis, phagocytosis, and receptor-mediated uptake mechanisms, leading to the accumulation of detectable levels of contracting agent in diseased brains.

Over the past decade, we have demonstrated in several mouse models of AD that liposomal nanoparticles administered intravenously cross the BBB into the CSF via the choroid plexus and microbleeds, carrying milligram quantities of imaging contrast payload per milliliter (Tanifum et al., 2012, 2014, 2016; Badachhape et al., 2020). We also showed that a variant of the probe designed to carry a hyperrelaxive Gd(III) MRI contrast payload targeted to Aβ plaques in the brain parenchyma shows high efficacy in separating Aβ^+^ from Aβ^−^ mice and is currently in clinical trials as the first MR-based molecular imaging agent for Aβ plaques in humans (Tanifum et al., 2016; Badachhape et al., 2020). We hypothesized that a variant of probe labeled with α-syn ligands (Figure 1A) can access and cross-link oligomeric α-syn in the CSF and interstitial fluid (ISF), forming cross-linked agglomerates of α-syn aggregates-nanoparticles in the process (Figure 1B). These agglomerates can then be internalized by both neurons and activated glial cells via similar mechanisms (conventional endocytosis, receptor-mediated endocytosis, and phagocytosis) as native oligomeric aggregates in the diseased brain (Figure 1C). However, the larger sizes of the agglomerates would make them much better substrates (>0.5 micrometers) (Flannagan et al., 2012; Galloway et al., 2019) and accelerate phagocytic uptake by activated microglia versus the endogenous oligomeric species (~200 nm). Overall, this will result in rapid accumulation of detectable levels of the contrast agent in diseased brains. A nontargeted probe in the same brain environment will not form any agglomerates and therefore will not show significant accumulation of the agent compared to the targeted variant. Similarly, a targeted probe in a brain without α-syn aggregates will fail to show significant accumulation of the probe due to the absence of a substate for agglomerate formation. To verify this hypothesis, we prepared an α-syn-targeted formulation (T) composed of the lipids shown in Figure 1A, with a recently reported α-syn selective ligand (Sun et al., 2022) as the targeting moiety, and a nontargeted control formulation (N). Using these two formulations and synthetic α-syn fibrils, we performed a series of *in vitro* experiments to verify nanoparticle/oligomeric α-syn agglomerate formation and cellular uptake of the ensuing agglomerates by neuronal, astrocyte and micrglia cell lines. Delayed *in vivo* MRI scans in A53T α-synuclein transgenic line M83 mice and controls including transgenic mice treated with N (TgN) and wild-type age-matched cohorts treated with T (WtT), following intravenous administration of the agent and *ex vivo* immunohistochemical analysis of brain tissue from treated mice were used to establish *in vivo* performance of the agent.

## 2 Methods

### 2.1 Fabrication of targeted liposomes (T)

α-Syn targeted liposomes were prepared using a standard hydration/extrusion protocol. Briefly, a lipid mixture containing HSPC, DSPE-mPEG(2000), cholesterol, Gd(III)DSPE-DOTA, and the XW-01-11 DSPE-PEG(3400)-conjugate (synthesis route in Supplementary Scheme S1) at a molar ratio of 32:2.5:40:25:0.5 in ethanol (600 μL) was stirred at 60–65°C until all solids dissolved to form a clear solution. DHPE-rhodamine dissolved in ethanol (1 mg in 200 μL) was added to the solution. The dissolved lipids were hydrated with histidine-buffered saline (HBS) (10 mM histidine, 140 mM NaCl, ~pH 7.6) at 60–65°C for 45 min. The hydrated lipid solution was extruded sequentially through 400 nm (5 passes) and 200 nm (8 passes) Nuclepore membranes at 60–65°C using a high-pressure extruder (Northern Lipids, Vancouver, BC, Canada) to form liposomes of the desired size. The liposomal suspension was dialyzed against histidine-buffered saline (HBS) using 300 kDa molecular weight cutoff membranes (Spectrum Laboratories Inc., CA, United States) to remove any unencapsulated lipids and ethanol. The nontargeted control formulation was prepared by the same procedure, but the lipid mixture did not include the targeting lipid conjugate or the DSPE-PEG(3400)-XW-01-11 conjugate. The nanoparticle size was determined by dynamic light scattering (DLS), and the concentrations of Gd(III) and total lipids in the final formulation were determined by inductively coupled plasma-optical emission spectrometry (ICP-OES).

### 2.2 Dynamic light scattering

To measure particle size, periodic samples from the extrusion process, and a final sample after diafiltration, were diluted in PBS and measured on a BI-90 goniometer based DLS system attached to a BI-9000 autocorrelation system. A 532 nm solid state laser was used as the light source, and the concentration of the sample adjusted until discriminated detection with a Hamamatsu photomultiplier tube at 90° yielded ~200 kcps. Correlation functions were measured using an exponentially spaced set of correlator bins, insuring at least 10 channels capturing the initial exponential drop of the correlation function, and 10 channels capturing the long-term decay. Correlation functions were averaged for 2 min for each sample, using a dust-discrimination algorithm that eliminated correlation function slices that showed long term correlation functions significantly higher than baseline, indicating contamination with large particle sizes characteristic of dust. The resulting averaged correlation functions were analyzed using the CONTIN algorithm, and the volume averaged distributions used to estimate a mean size and standard deviation. All distributions were practically unimodal (> = 99% of volume in the main peak).

### 2.3 Transmission electron microscopy

Holey Carbon Film grids (Quantifoil R 1.2/1.3 + 2 nm Cu 200 mesh) were glow discharged at 15 μA for 10 s. 3.5 μL of sample (the solution included 0.5 μM fibrils, or 10 μM liposomes with 0.5 μM fibrils) was applied to the carbon-coated side of the glow discharged grid and allowed to set for 2 min. While waiting for the sample to set, for each grid, two 20 μL drops of distilled water and two 20 μL drops of 2% uranyl acetate (UA) solution that was filtered the same day were placed on a piece of parafilm. After 2 min, the sample was dabbed off very gently with a piece of filter paper. The first droplet of water was applied to the sample and then dabbed off. The process was repeated with the second drop of water. Next, the first drop of stain (UA) was applied and after 10 s it was dabbed off. Finally, the second drop of stain (UA) was applied and dabbed off after for 1 min. The grids were then air dried for 5–10 min and placed in a grid box. The grid box was then placed in a desiccator overnight to ensure the grids were completely dry before imaging. Images were captured using a Jeol 2100 Electron Microscope (Beam Voltage Value: 200 kV; Beam Current Value: 106–112 μA; Gun: LaB6; Gun Spot Size#3; Condenser Lens Aperture: #1 (200 μm); Objective Lens Aperture: #1 (60 μm); Exposure Time:1 s; Magnification: 8,000 and 25,000).

### 2.4 α-synuclein fibrils preparation

Fibrils were prepared from α-synuclein peptide (R-peptide, Bogart, GA) as previously described (Sun et al., 2022). Briefly, the α-syn peptide (0.5 mg) was suspended in 0.2 mL water and 0.2 mL phosphate buffer (10 mM, pH 7.5) and centrifuged (18,000 g) for 5 min to remove any soluble materials. The ensuing pellet was resuspended in 2.5 μL of 300 mM MnCl_2_ and stirred at 40°C in an incubator for 7 days. The resulting hazy solution was spun down at 21,000 rcf for 6 min. The supernatant was decanted and the pellet resuspended in 200 μL PBS buffer (pH = 7.4) to obtain a final concentration of peptide 129.6 μM in the form of fibrils.

### 2.5 HMC-3 and T98G cell culture

The human microglial clone 3 cell line (HMC-3) and T98G cells were purchased from American Type Culture Collection (ATCC, Manassas, VA, United States). HMC-3 and T98G cells were cultured in Eagle’s minimum essential media (EMEM, ATCC) supplemented with 10% FBS (Sigma–Aldrich, St. Louis, MO, United States) and 100 units/mL (U/mL) penicillin/streptomycin (Invitrogen, Carlsbad, CA, United States) and incubated in a humidified atmosphere (5% CO_2_) at 37°C.

### 2.6 Culture and differentiation of the SH-SY5Y cells

#### 2.6.1 Cell culture

SH-SY5Y cells were purchased from American Tissue Culture Collection (ATCC, Manassas, VA, United States). The culture media constituted Dulbecco’s modified Eagle’s medium (DMEM, Thermo Fisher Scientific, Waltham, MA, United States) supplemented with fetal bovine serum (FBS, Thermo Fisher Scientific) and 100 units/mL (U/mL) penicillin/streptomycin (Invitrogen, Carlsbad, CA, United States). Cells were suspended in the media and incubated in a humidified atmosphere (5% CO_2_) at 37°C.

#### 2.6.2 Cell differentiation

The cultured cells were differentiated following the procedure described by the Shipley et al. (2016). Differentiation media A constituted 2.5% FBS and 10 μM retinoic acid; differentiation media B constituted 1% FBS and 10 μM retinoic acid. Cells (2 × 10^4^ cells/chamber) were seeded in 8 chamber culture slides (Falcon^™^ 354118) and allowed to adhere and grow for 1 day. The media was removed by gentle aspiration and replaced with 250 μL differentiation media A in each chamber. The cells were then incubated in a humidified atmosphere (5% CO_2_) at 37°C for 2 days. The procedure repeated every 2 days, and cell growth was monitored everyday using a microscope. On day 5, differentiation media A was removed by gentle aspiration and replaced with 250 μL of differentiation media B in each chamber. Cells appeared differentiated on day 9 and were treated with either T or N (10 μM lipid). After 1.5 h of incubation, the supernatant was removed, and the cells washed with PBS three times. The cells were fixed in 4% paraformaldehyde for 15 min and permeabilized by washing three times for 5 min with PBS containing 0.1% Triton X-100. The cells were then blocked with 3% normal goat serum for 30 min, washed with PBS, and incubated with phalloidin 488 (1:100, ab176753, Abcam) for 1 h. This was followed by another wash with PBS and then incubation with Hoechst (33342, Thermo Fisher Scientific) at room temperature for 7 min. Finally, the cells were washed with PBS three times, coverslipped, and imaged under an Olympus IX81 microscope [DAPI (DAPI 405 nm), phalloidin (Alexa Fluor 488), T/N (Rhodamine red-X)].

### 2.7 Inflammatory potential

(ELISA MAX^™^ Deluxe Set Human TNF-α; ELISA MAX^™^ Deluxe Set Human IL-1β; ELISA MAX^™^ Deluxe Set Human IL-6; Biolegend). The experiments were performed as described in the technical data sheet.

### 2.8 Cell uptake studies

To label α-syn fibrils with extrinsic fluorophores, 0.5 mL of α-syn fibrils were incubated with 5 molar equivalents of Alexa Fluor^™^ 750 NHS-Ester (succinimidyl ester) fluorophore for 1 h at room temperature (Scheiblich et al., 2021). The reaction was quenched by the addition of 1 mM Tris, pH 7.5, and the unreacted fluorophore was removed by two cycles at 15,000 g for 10 min and resuspension of the pellets in PBS.

HMC-3, T98G, or SH-SY5Y cells (2 × 10^4^ cells/chamber) were seeded on 8 chamber culture slides (Falcon^™^ 354,118) and allowed to adhere overnight. The cells were treated with 1 μM α-syn fibrils or Alexa Fluor^™^ 750-labeled α-syn fibrils and incubated for 15 min, followed by the addition of T or N (10 μM lipid). After 1.5 h of incubation, the supernatant was removed by pipetting, and the cells were washed with PBS three times. The cells were fixed in 4% paraformaldehyde for 15 min and permeabilized by washing three times for 5 min with PBS containing 0.1% Triton X-100. The cells were then blocked with 3% normal goat serum for 30 min, washed with PBS, and incubated with phalloidin 488 (1:100, ab176753, Abcam) for 1 h. This was followed by another wash with PBS and then incubation with Hoechst (33342, Thermo Fisher Scientific) at room temperature for 7 min. Finally, the cells were washed with PBS three times, coverslipped, and imaged under an Olympus IX81 microscope [DAPI (DAPI 405 nm), phalloidin (Alexa Fluor 488), T/N (Rhodamine red-X), fibrils signal (Alexa Fluor 750)].

### 2.9 Animal studies

The A53T α-synuclein transgenic line M83, commonly referred to as M83 transgenic mice, expresses mutant human A53T alpha-synuclein under the direction of the mouse prion protein promoter. Some mice homozygous for the transgenic insert develop a progressively more severe motor phenotype at 8 months of age, but on average, the phenotype fully manifests at 14–15 months of age. Along with the motor impairment phenotype, they also develop age-dependent intracytoplasmic alpha-synuclein aggregate inclusions. Immunohistochemistry analysis of mutant mice between eight and 12 months of age revealed widely distributed alpha-synuclein inclusions, with dense accumulation in the spinal cord, brainstem, cerebellum, and thalamus. Pathology in older mice may also spread to other parts of the CNS, paralleling features of the disease in humans. For this proof-of-concept study, we chose two different age groups of transgenic mice in which the pathology is known to be fully established: 13- to 15-month-old mice, representing intermediate stages of the disease, and 16+-month-old mice, representing late stages of the disease (Giasson et al., 2002; Lee et al., 2002).

All animal studies reported in this paper were conducted under study-specific protocols approved by the Institutional Animal Care and Use Committee (IACUC) at Baylor College of Medicine. In all animal experiments, 6 mice were used in each test or control group. Animals were anesthetized using 2% isoflurane in an induction chamber followed by maintenance on 1–1.5% isoflurane delivered using a nose cone setup during all injection and imaging procedures. The tail vein was catheterized for injection liposomes (dosage, 0.1 mmol Gd/kg body weight).

### 2.10 Pharmacokinetics and biodistribution study

Nine- to twelve-week-old C57BL/6 mice were used in this study. MRI was performed on a 1 Tesla permanent magnet scanner (M7, Aspect Imaging, Shoham, Israel). Images were acquired pre-contrast to serve as a baseline, followed by intravenous administration of the agent via the tail vein at a dose of 0.1 mmol Gd/kg. Post-contrast images were acquired at the following time points: 0 h (within 5 min after injection), 1 h, 2 h, 4 h, 8 h, 1 day, 2 days, 4 days, 7 days, and 14 days. Images were analyzed in Osirix (version 5.8.5, 64-bit). Regions of interest (ROIs) were drawn in the blood compartment (inferior vena cava), and organs (liver, spleen, kidneys, and brain) were selected to generate signal-time curves. The values are reported as T1 relaxation rates (R1) in units of s^−1^. The blood half-life (t1/2) was determined by fitting the signal change in the IVC to a model of exponential decay and was found to be 20.23 ± 0.59 h. Organs (including liver, spleen, kidney, and brain) were harvested 14, 28, and 60 days after agent administration, weighed, and digested with nitric acid to measure residual Gd(III) content using inductively coupled plasma-mass spectrometry (ICP-MS).

### 2.11 Tissue Gd(III) content by inductively coupled plasma-mass spectrometry

Gd(III) concentration in tissue samples was quantified using ICP-MS (Agilent, CA, United States). Wet tissue (~100 mg) was digested in 90% concentrated HNO_3_ (~750 μL) at 90°C for 10–15 min. The digested sample was diluted in deionized (DI) water, vortexed vigorously and centrifuged at 3,500 rpm for 15 min. The supernatant was separated and further diluted as needed to ensure Gd concentrations fell within the range of calibration standards (1–500 ppb). Quality control samples (50 and 100 ppb) were included at the start, middle and end of analysis runs.

### 2.12 *In vivo* MRI

Mice underwent pre-contrast baseline scans followed by intravenous administration of the nanoparticle MR contrast agents [α-syn-targeted formulation (T) or nontargeted control formulation (N)] via the tail vein at a dose of 0.1 mmol Gd/kg body weight. Delayed post-contrast MRI was performed 4 days after contrast agent injection. Pre-contrast and delayed post-contrast MR images were acquired using a T1-weighted spin-echo (T1w-SE) sequence and a fast spin-echo inversion recovery (FSE-IR) sequence with the following parameters: *SE parameters*: TR = 600 ms, TE = 11.5 ms, slice thickness = 1.2 mm, matrix = 192 × 192, FOV = 30 mm, slices = 16, NEX = 4; *FSE-IR parameters*: TR = 13,500 ms, TE = 80 ms, TI = 2000 ms, slice thickness = 2.4 mm, matrix = 192 × 192, FOV = 30 mm, slices = 6, NEX = 6. Coil calibration, RF calibration, and shimming were performed at the beginning of the study for each subject. Pre-contrast scans provide a baseline for calculating signal enhancement from resulting post-contrast scans (Badachhape et al., 2020). The test group within each age group consisted of six transgenic mice injected with the targeted formulation (TgT), while the control groups consisted of six transgenic mice injected with the nontargeted formulation (TgN) and six wild-type mice injected with the targeted formulation (WtT). At 4 days post-contrast administration, when all unbound contrast agent had cleared from the blood pool, the animals were reimaged using the same scan parameters as in the pre-scans to determine any changes in the brain. Image analyses and signal quantification were performed with Osirix software (Pixmeo, Geneva, Switzerland). For quantitative signal analysis, signals from ~1.2 mm thick coronal slices at three different positions around bregma 3.92 mm (olfactory bulb), bregma 0 (parietal-temporal lobe), and bregma −6.64 mm (brain stem) were used to represent signals for the respective brain regions. The signal beyond bregma −6.64 mm showed some inconsistencies attributed to magnetic field inhomogeneity at this position in the head coil of the magnet.

Two-standard deviations above the mean variation within wild-type control animals were used as the cutoff signal intensity for identifying α-syn-positive animals. Qualitative and quantitative analyses of the MR images were performed in OsiriX (version 5.8.5, 64-bit, Pixmeo SARL, Geneva, Switzerland) and MATLAB (version 2015a, MathWorks, Natick, MA).

### 2.13 Histology analysis

After the last MRI scan, the mice were perfused with PBS and 4% formalin, after which the brains were excised and stored in 30% sucrose solution until sectioning for histological analysis.

#### 2.13.1 Immunofluorescence

The brains were embedded in Tissue-Tek O.C.T. and kept in liquid nitrogen for 30 min. The embedded tissue was sliced into 30 μm thick sections with Lecia Biosystems Cryostats at −20°C. As exemplified by the procedure for Lewy pathology, immunofluorescence studies were performed as follows: tissue sections were washed twice with PBS, followed by antigen retrieval with citric acid buffer (pH ~ 6.0)/microwave for 30 min. The sections were then washed with PBS three times and permeabilized with 0.1% Triton-X 100 for 10 min, followed by washing with PBS (twice). The tissue was incubated with 10% normal goat serum at room temperature for 1 h, followed by incubation with a primary antibody, an anti-alpha-synuclein (phospho S129) antibody [EP1536Y] (ab51253, Abcam) (1:500 in 1% goat serum), overnight at 4°C. The tissue was washed with PBS three times and incubated for 2 h at room temperature with Alexa Fluor 750-labeled secondary antibody (1:200 in PBS). After being washed with PBS two times, the tissue was incubated with Hoechst (33342, Thermo Fisher Scientific) at room temperature for 7 min. The sections were washed with PBS three times, coverslipped, and imaged under an Olympus IX81 microscope [DAPI (DAPI 405 nm), T/N (Rhodamine red-X), pS129-α-syn/IBA-1/NF-F/GFAP (Alexa Fluor 750)]. The same procedure was used for anti-IBA-1 (IBA-1 Polyclonal Antibody, Unconjugated, Host: Rabbit/IgG, Thermo Scientific), anti-GFAP [anti-GFAP Mouse Monoclonal Antibody (clone: 2E1. E9), Biolegend] and anti-NF-H [antibody NF-H, Phosphorylated (SMI 31), Biolegend] staining.

#### 2.13.2 DAB staining

The brain sections were washed with PBS two times and subjected to antigen retrieval in citric acid buffer (pH ~ 6.0)/microwave radiation for 30 min. The sections were washed with PBS two times, after which endogenous peroxidase activity was blocked by incubation with 0.3% H_2_O_2_ for 10 min. Next, the sections were washed with PBS three times and permeabilized with 0.1% Triton-X 100 for 10 min, followed by washing with PBS three times. The tissue was incubated with 10% normal goat serum at room temperature for 1 h, followed by incubation with anti-alpha-synuclein (phospho S129) antibody [EP1536Y] (ab51253, Abcam) (1:500 in 1% goat serum) overnight at 4°C. The sections were washed with PBS three times and then incubated with a biotinylated secondary antibody (1:200) for 30 min, followed by incubation with an ABC Elite Kit (1:100 each A and B mixed) for 1 h. The tissue was washed with PBS three times and incubated in 3,3′-diaminobenzidine solution and monitored for staining (approximately 1 to 3 min), and the reaction was stopped with distilled water. The tissue was then dehydrated and cleared with ethanol and xylene and imaged under a Leica DFC365 FX microscope (CCD Microscope Camera).

### 2.14 Statistical analyses

Statistical analysis for the *in vitro* cell experiments was performed through one-way ANOVA with multiple pairwise comparisons between groups using Fisher’s least significant difference (LSD) test. Statistical analysis of *in vivo* experimental data were performed through the Kruskal–Wallis method with multiple pairwise comparisons using the Bonferroni method.

## 3 Results

### 3.1 Nano scavenger design and formulation

The design of the MR-sensitive oligomeric α-syn-targeted nano scavenger was based on highly T1-relaxive liposomal Gd(III), in which the macrocyclic Gd(III)-DOTA chelate is expressed on the lipid bilayer (Figure 1A). It has been demonstrated previously, that this formulation generates Gd(III) solutions with approximately five times the T1 molar relaxivity of conventional Gd(III)-DOTA solutions (Tanifum et al., 2016). Commercially available DHPE-rhodamine was included in the formulation as a fluorescent reporter for confocal microscopy imaging experiments. mPEG(2000)-DSPE was used to provide standard stealth properties. The targeting moiety, based on one of our previously reported α-syn aggregate-binding indanones (XW-01-11) (Sun et al., 2022) was synthesized as shown in Supplementary Scheme S1. Both the targeted test (T) and nontargeted control (N) formulations were prepared using standard liposome formulation protocols. DLS measurements of T showed particles with a hydrodynamic diameter of 185.2 ± 5.3 nm, and ICP-MS data showed a final lipid concentration of 88.4 ± 1.2 mM. The hydrodynamic diameter of N was 151.1 ± 2.2 nm, and the lipid concentration was 84.0 ± 2.8 mM.

### 3.2 *In vitro* verification and characterization of cross-linked oligomeric α-synuclein/nano scavenger agglomerate formation

**Figure 2:**
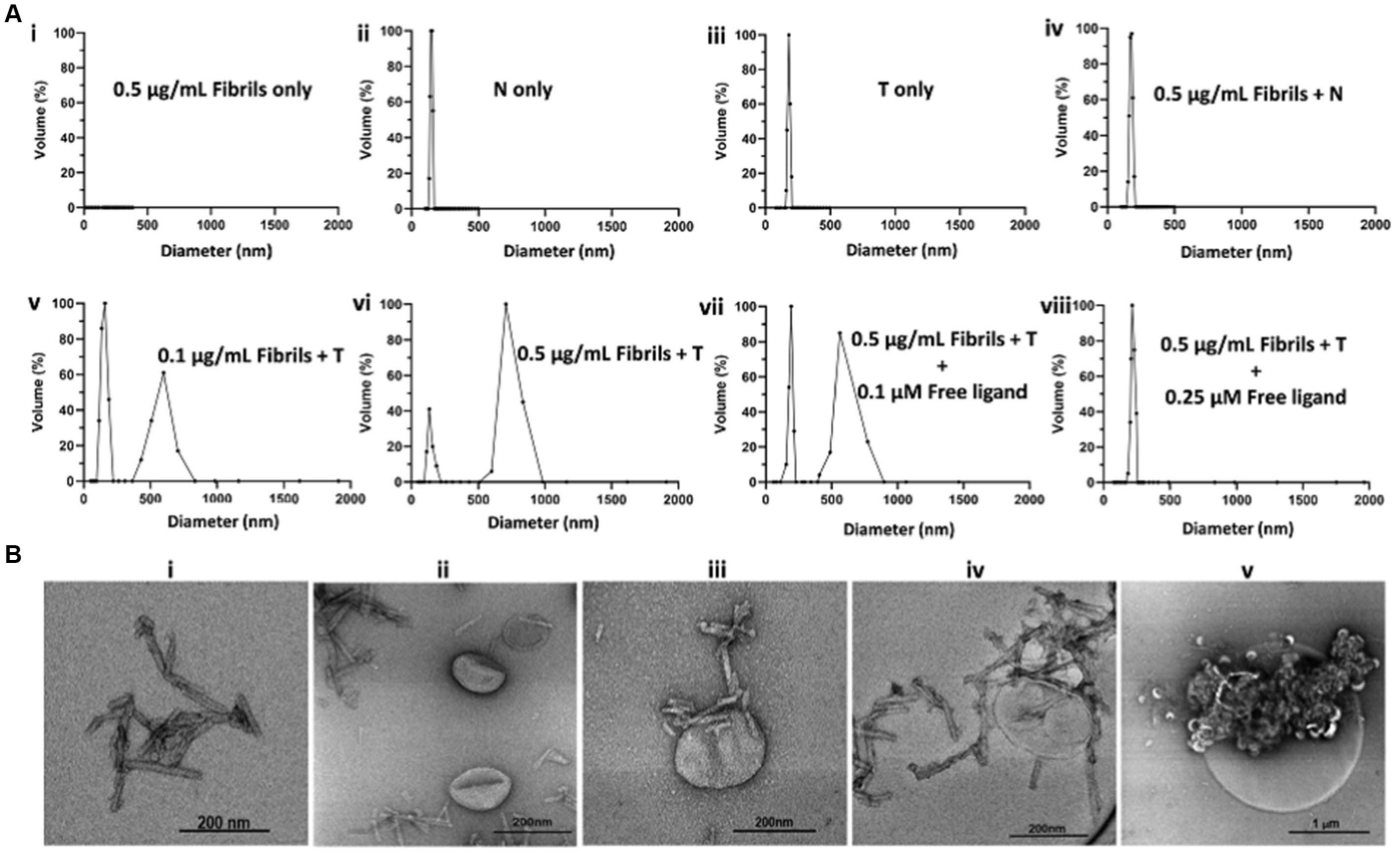
*In vitro* characterization of T/α-syn fibril agglomerate formation by DLS and TEM. All DLS experiments were run at a lipid concentration of 10 μM for both T and N. **(A)** DLS profiles of solutions of T or N at different fibril concentrations: **(Ai)** 0.5 μg/mL solution of synthetic α-syn fibrils shows no DLS signal. **(Aii)** Solution of N shows a particle distribution of ~150 nm. **(Aiii)** Solution of T shows a particle distribution of ~185 nm. **(Aiv)** 0.5 μg/mL solution of incubated with N shows no major change in particle distribution. **(Av)** 0.1 μg/mL solution of fibrils incubated with T shows the original particle population with a hydrodynamic diameter of ~185 nm and a new population with a hydrodynamic diameter of ~600 nm, attributed to T/fibril agglomerate formation. **(Avi)** Increase in fibril concentration from 0.1 μg/mL to 0.5 μg/mL results in an increase in hydrodynamic diameter of larger particles to ~750 nm, attributed to an increase in cross-linking at a higher fibril concentration. **(Avii)** Maintaining the fibril concentration at 0.5 μg/mL but introducing a free fibril-binding ligand at a 0.1 μM concentration results in an increase in the ~180 nm volume fraction and a decrease in the ~750 nm volume fraction. **(Aviii)** Saturation of ligand binding sites on the fibrils with 0.25 μM free ligand results in no agglomerate formation. **(B)** TEM images of α-syn fibrils exposed to T. **(Bi)** TEM image of α-syn fibrils. **(Bii)** TEM image of N incubated with α-synuclein fibrils. **(Biii)** TEM image of α-synuclein fibrils bound to a single T particle from a solution of T incubated with fibril. **(Biv)** TEM image of a nanometer size cluster of T bound to fibrils. **(Bv)** TEM image of micron size T/fibrils agglomerate formed in a 0.5 μg/mL solution of synthetic α-fibrils incubated with T.

To determine whether T will form agglomerates upon exposure to oligomeric α-syn aggregates, we performed a series of *in vitro* experiments in which T was exposed to synthetic α-syn fibrils under different conditions, after which the resulting products were characterized by DLS and transmission electron microscopy (TEM) (Figure 2). Synthetic α-syn fibrils were prepared as previously described (Sun et al., 2022). A solution of fibrils (0.5 μg/mL) showed no DLS signal (Figure 2Ai), while a solution of N at a lipid concentration of 10 μM showed particles with a hydrodynamic diameter of ~150 nm (Figure 2Aii), and a solution of T (targeted formulation) at a lipid concentration of 10 μM showed particles with a hydrodynamic diameter of ~185 nm (Figure 2Aiii). We attributed the apparent difference in particle size between N and T to the PEG(3400) tether bearing the targeting ligands on the surface of the T particles. Otherwise, the actual vesicle size is expected to be similar for both formulations since they were subjected to the same extrusion protocol. The DLS profile of solutions in which the lipid concentration of N was maintained at 10 μM but with different fibril concentrations showed a very slight shift in the hydrodynamic diameter of the particles in solution, as exemplified by Figure 2Aiv (fibril concentration = 0.5 μg/mL). When fibrils (0.1 μg/mL final concentration) were added to a 10 μM lipid solution of T, two distinct particle populations were observed: one consisting of the original particles and a second population of larger particles, some with a diameter of up to a micron (Figure 2Av). An increase in the concentration of fibrils (as exemplified by the profile in Figure 2Avi, fibril concentration = 0.5 μg/mL) led to an increase in the population of larger particles, suggesting agglomerate formation upon exposure of T to fibrils but not N.

To ascertain whether agglomerate formation was due to T surface ligands binding to fibrils, blocking experiments were conducted in which T and fibrils were co-incubated with the free ligand at different concentrations. As exemplified by the DLS profiles in Figures 2Avii,viii, co-incubation with 0.1 μM free ligand reduced the size of the larger particle population, and complete saturation of binding sites on the fibrils with 0.25 μM free ligand in solution resulted in no change in particle distribution. Taken together, these data suggest *in vitro* agglomerate formation upon exposure of T to fibrils. A full panel of DLS profiles from the fibril dilution and blocking studies is shown in Supplementary Figure S1. To further confirm the nature of the T/fibril interactions, samples from solutions of fibrils and solutions of fibrils incubated with T were assessed by transmission electron microscopy (Figure 2B; Supplementary Figure S2). Figure 2Bi represents a sample TEM image of fibrils seen on grids prepared from solutions containing fibrils only, while Figure 2Bii represents a sample image from a grid prepared from a solution of N incubated with fibrils. As shown in Figures 2Biii–v, the TEM images of the grids prepared from sample solutions of T incubated with fibrils reveal a variety of agglomerate species.

### 3.3 *In vitro* accelerated cellular uptake of the agglomerates

The three key cellular species associated with the spread of α-syn pathology in PD have been identified as neurons, astrocytes, and microglia (Mavroeidi and Xilouri, 2021).

**Figure 3:**
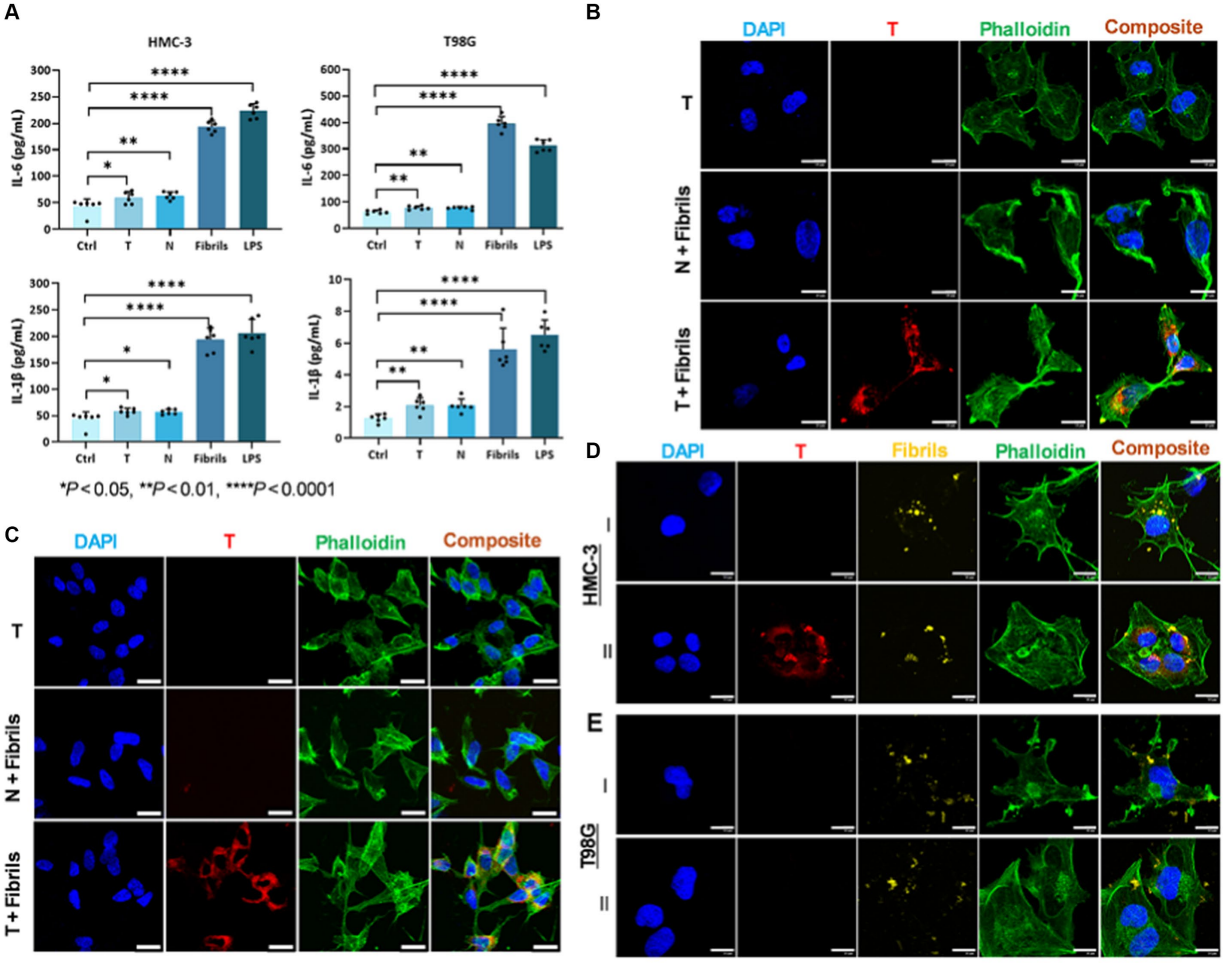
*In vitro* exposure of T to neuronal and glial cell lines in the presence of α-fibrils results in accelerated internalization of T. **(A)** Exposure of HMC-3 and T98G cells to α-syn fibrils (1.0 μM) results in the release of elevated levels of cytokines, including IL-6 and IL-1β, confirming activation. **(B)** Confocal microscopy images of HMC-3 cells (microglial cell line) incubated with T alone or N plus fibrils show no apparent cell interaction with the nano scavengers (Rhodamine, red fluorescence) within 1.5 h of incubation, while cells incubated with T and fibrils show a conspicuous red signal. Composite images of nuclei (stained with DAPI, blue) and the cytoskeleton (stained with phalloidin-488 nm, green fluorescence) showing that the rhodamine signal is cytosolic. **(C)** A neuronal cell line (SH-Sy5y cells differentiated with rhetinoic acid) subjected to the same evaluation protocol showed that the cells also internalized T in the presence of fibrils. **(DI)** HMC-3 cells incubated with labeled fibrils (Alexa FluorTM-750 nm) showed fibril internalization in the absence of T. **(DII)** HMC-3 cells incubated with T and labeled fibrils showed internalization of both species. More importantly, T and labeled fibrils signals colocalized in the cytosol. **(EI)** T98G cells incubated with labeled fibrils did not show the clear internalization of T as observed in their HMC-3 counterparts. **(EII)** T98G cells incubated with T and labeled fibrils showed neither avid uptake nor colocalization of signals from the two species in the cytosol. Statistics: one-way ANOVA (pairwise—Fisher’s LSD): ^*^p < 0.05, ^**^p < 0.01, and ^****^p < 0.0001. Scale bar = 20 μm.

To verify the potential of these cells to internalize T/α-syn agglomerates as hypothesized, we performed *in vitro* cell experiments in which three different cell lines—SH-SY5Y differentiated with retinoic acid (a neuronal cell line) (Xicoy et al., 2017), T98G (an astrocyte cell line), and HMC-3 (a microglial cell line)—were exposed to preformed α-syn fibrils and T under various conditions. First, to ensure that the chosen glial cell lines became activated upon exposure to the fibrils, they were each incubated with fibrils for 24 h, and the incubation media was assayed for cytokines. The controls included cells treated with liposaccharide (LPS) and untreated cells. T and N—were also subjected to the same protocol to evaluate their inflammatory potential. The results (Figure 3A) show that both the HMC-3 and T98G cells exposed to the fibrils exhibited significant cytokine release relative to the untreated controls and the two formulations. Following this confirmation, the interaction of each of the three different cell lines upon exposure to T and the fibrils was assessed. Controls included cells incubated with fibrils and N, and cells incubated with T alone. As exemplified by the confocal microscopy images in Figure 3B, HMC-3 cells incubated with fibrils and T showed interactions with T (rhodamine signal) within 1.5 h of incubation, while cells incubated with either T only or N and fibrils showed no apparent interaction with the probe. Composite images generated by merging signals from nuclear (DAPI) and cytoskeletal (phalloidin-488 nm) stains show that the red signal is cytosolic. Taken together with the control data, these findings suggest that the presence of fibrils is a requisite for T internalization. When subjected to treatment protocol as the HMC-3 cells, SH-SY5Y cells differentiated with retinoic acid also showed internalization of T in samples treated with T and fibrils but not in the controls (Figure 3C). When subjected to the same protocol, the astrocyte cell line did not show avid internalization of the probe, like its neuronal and microglial counterparts. To gain more insights into the role of fibrils in T internalization, the fibrils were labeled with a fluorescent tag (Alexa Fluor^™^-750 nm) and incubated with HMC-3 cells in the absence of T. As shown in Figure 3DI, within 1.5 h of incubation, the HMC-3 cells showed fibril internalization (yellow fluorescence) in the absence of T. When they were incubated with both T and labeled fibrils (Figure 3DII), both species were internalized. More importantly, all the prominent labeled fibril signals were colocalized with the strong and bright signal points originating from T in the composite images. This finding suggests the formation and internalization of both large and small agglomerates during the process. The bright signal spots were attributed to agglomerates with a larger hydrodynamic diameter, while the more diffuse signal points were attributed to smaller agglomerate species resulting from encounters between T and fibrils in the incubation mixture. Unlike their HMC-3 counterparts, the T98G cells (Figure 3E) neither showed no convincing evidence of internalization of the fibrils, either by themselves (Figure 3EI) or when co-incubated with T (Figure 3EII).

### 3.4 *In vivo* pharmacokinetics and biodistribution of nano scavengers

To confirm that T exhibits an *in vivo* profile similar to other liposome formulations, 9- to 12-week-old C57BL/6 mice were administered the agent via tail vein injection. T1 maps were collected around the brain, inferior vena cava (IVC), kidneys, liver, and spleen at different time points over 14 days following intravenous administration. By day 14, the signal in the blood and other organs had returned to baseline levels, except in the liver and spleen. Any residual Gd(III) in these organs was further assessed over a 60-day period using ICP-MS. Data from the T1 maps and ICP-MS analysis are presented in Supplementary Figure S3. Taken together, the data suggest that the formulation has a systemic circulation half-life of 20.2 ± 0.6 h and is cleared via the monocyte-phagocyte system pathway, consistent with previously reported results for pegylated liposomes (Immordino et al., 2006).

### 3.5 Noninvasive interrogation of pathologic α-syn accumulation and reactive gliosis

**Figure 4:**
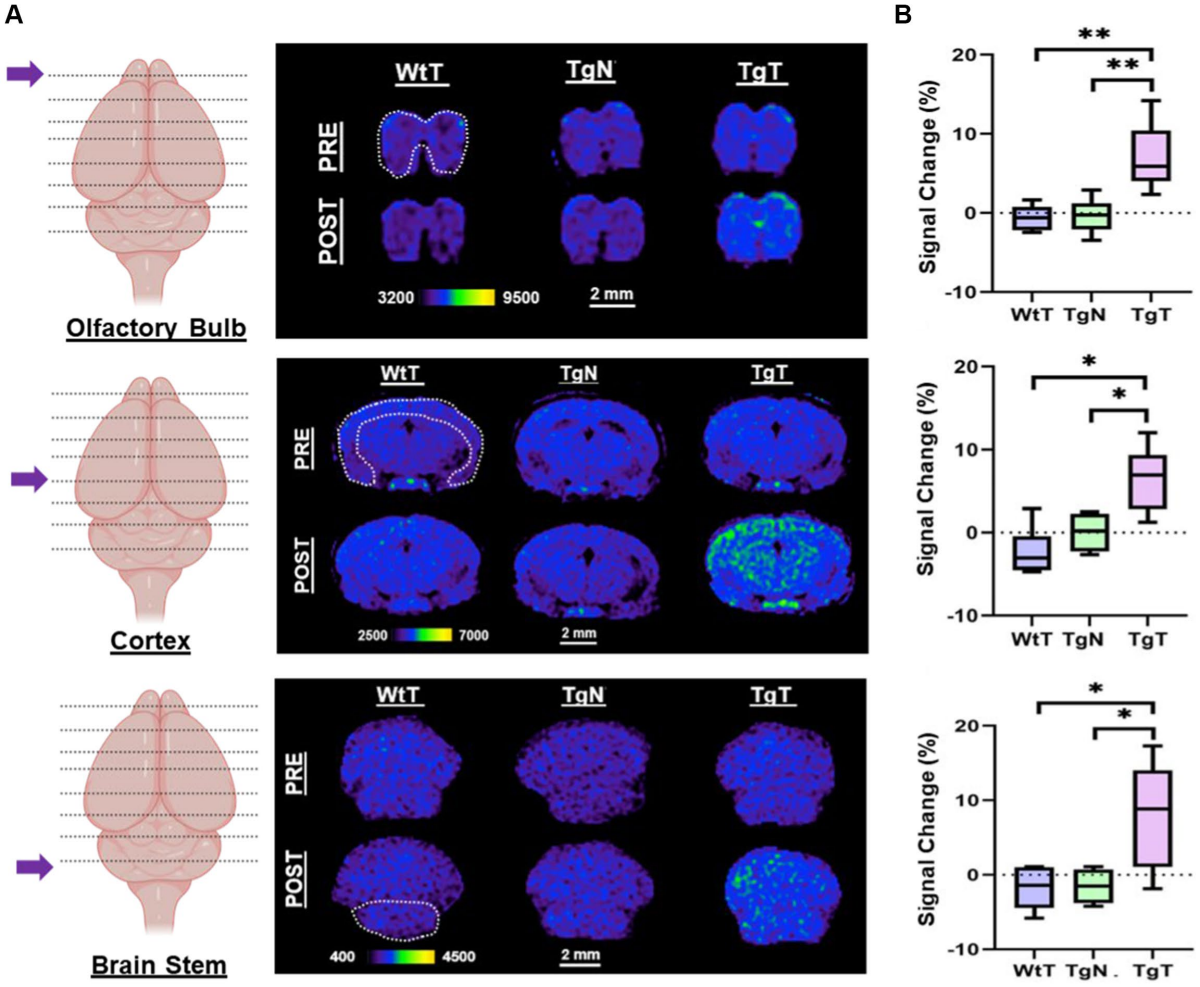
*In vivo* T1-weighted MR images pre- and post-contrast administration demonstrated statistically significant signal enhancement in M83 α-syn transgenic mice treated with T compared with controls. **(A)** A comparison of 1.2 mm thick slices from T1-weighted spin–echo (T1w-SE) images at 4 days post nano scavenger administration around the olfactory bulb, cortex, and brainstem of 16+ month old TgT (*N* = 6) against TgN (*N* = 6), and WtT (*N* = 6) mice demonstrated signal enhancement in all the brain regions in TgT mice compared with the TgN and WtT cohorts. **(B)** Box and whisker plots of percent signal enhancement in all three brain regions show statistically significant mean enhancements in TgT against TgN and WtT. Statistics: Kruskal–Wallis (pairwise—Bonferroni): ^*^p < 0.05 and ^**^p < 0.005.

As shown in the pseudo-colored images from a 19-month-old mouse (Figure 4A), signals (pre- and post-contrast scans) from each brain region were windowed similarly for both the test and control groups (TgT, TgN, and WtT). Visual observation suggested greater signal intensity (green color) in the post-contrast images of TgT than in those of TgN and WtT. The signal change (%) between pre-contrast and delayed post-contrast images for each brain region was quantified by the integration of signals in each region of interest, as shown by the white dotted lines. Box and whisker plots of the signal change in test animals versus controls (Figure 4B) show that TgT mice exhibit significant signal enhancement between post-contrast T1w-MRI and pre-contrast MRI relative to their WtT or TgN counterparts. It should be noted that strong regional differences were observed in the olfactory bulb and parietal-temporal lobe, while the brainstem regions showed greater variance. Despite this variance, all three regions demonstrated strong *in vivo* MR signals resulting from retention of the Gd(III)-loaded targeted agents. A similar but less pronounced signal enhancement profile was observed in the brains of 13- to 15-month-old TgT cohorts against controls (Supplementary Figure S4).

### 3.6 Correlations between *in vivo* MR signal enhancement, nano scavenger signal, and phospho-S129-α-synuclein (pS129-α-syn) immunoreactivity

**Figure 5:**
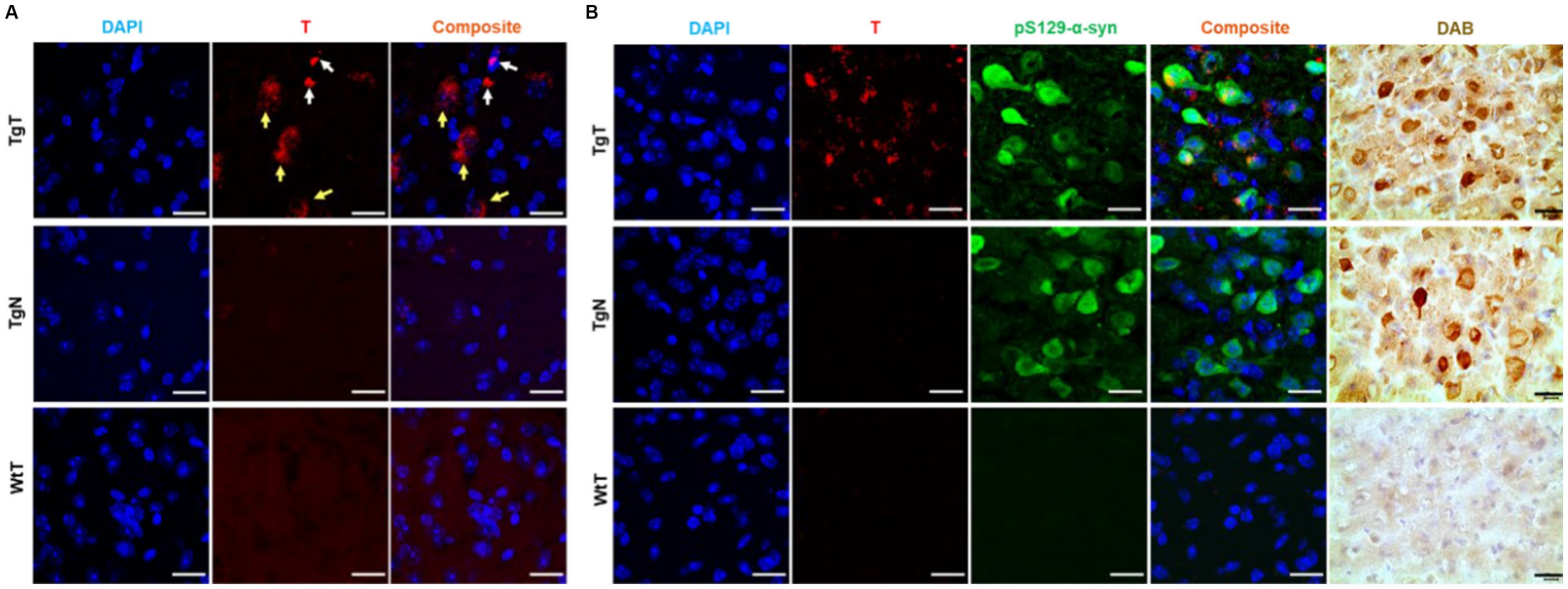
Confocal microscopy imaging demonstrated a correlation between accumulation of nano scavengers and Lewy pathology in the entorhinal cortex. **(A)** Tissue sections from TgT mice showed strong fluorescence in the rhodamine channel compared to very little to no fluorescence in tissue from TgN and WtT controls. Closer examination revealed that part of the red fluorescence emanated from compact structures (white arrows) and the other part from diffuse structures (yellow arrows). **(B)** Anti-pSer129-α-syn-stained cortical tissue sections from a 19-monthold TgT mouse shows colocalization of rhodamine fluorescence with Lewy pathology. Contiguous slides subjected to the DAB protocol using pS129-α-syn (tailored for high specificity) confirmed the observed structures as Lewy pathology. TgN control shows no rhodamine signal due to no accumulation of the nontargeted agent but strong presence of Lewy pathology due to the mouse’s genotype while WtT controls show neither rhodamine signal nor the presence of Lewy pathology. Scale bar = 20 μm.

To confirm that the observed *in vivo* MR signal enhancement in TgT mice was due to an interplay between α-syn aggregates and cellular participants, brain tissue from all the mice was assessed via immunohistochemical staining and confocal microscopy imaging, following MRI. First, signals from the rhodamine (fluorescent reporter on nanoparticles) channel in confocal microscopy images from TgT tissue were compared against images from TgN and WtT control tissues. As shown in Figure 5A, TgT brains showed fluorescence in the rhodamine channel, suggesting the presence of T in the tissue. On closer examination, the fluorescence appeared to emanate from two different sources: one that is very bright (white arrows), suggesting a more compact structure with a high concentration of the fluorophore, and the other that appeared slightly blurred (yellow arrows), suggesting somewhat more diffuse structures. A composite image generated by merging the rhodamine signal with a DAPI-stained nuclear image of the tissue showed that all the rhodamine signals were in very close proximity to the nuclei, suggesting that the signal could be cytosolic. Very little to no rhodamine signal was observed in the images of the control tissue, consistent with the little to no positive signal enhancement in the *in vivo* MR images and suggesting that the signal enhancement was due to T accumulation in the tissue.

To assess the relationship between the observed *in vivo* MR signal enhancement and Lewy pathology, the imaged brain tissue sections were stained with an antibody against phospho-S129-α-synuclein (pS129-α-syn) to detect aggregated α-syn species (Delic et al., 2018; Lashuel et al., 2022). All brain regions that displayed *in vivo* MRI signal enhancement (olfactory bulb, parietal-temporal lobe, and brain stem) also showed Lewy pathology in association with the T signal. As exemplified by images of cortical tissue sections from a 19-month-old TgT mouse (Figure 5B), the tissue sections showed signals from T, in association with strong anti-pS129-α-syn staining throughout the entorhinal cortex (green fluorescence). Lewy pathology in the cortex and olfactory bulb is not widely reported in this model, likely because most studies have used mice younger (2 to 12 months old) than those used in this study. To confirm that the observed green fluorescence was indeed Lewy pathology, contiguous tissue sections were stained with a pS129-α-syn DAB staining protocol, which can be tailored to stain pathological α-syn with high specificity and no cross-reactivity with other proteins (Beach et al., 2008). Bright field images of DAB-stained tissue (brown fluorescence) showing intense staining of the characteristic punctate structures of Lewy bodies on the TgT tissue. Although the TgN controls did not show any strong signal from T, consistent with little to no *in vivo* MRI signal enhancement, they showed strong anti-pS129-α-syn reactivity, which is consistent with their genotype. Images highlighting correlation between T and Lewy pathology in the olfactory bulb and brainstem as well as distribution of the pathology in mice in other age groups are shown in Supplementary Figures S5–S7.

### 3.7 Correlations between *in vivo* MRI signal enhancement, nano scavenger signal, and IBA-1 immunoreactivity

**Figure 6:**
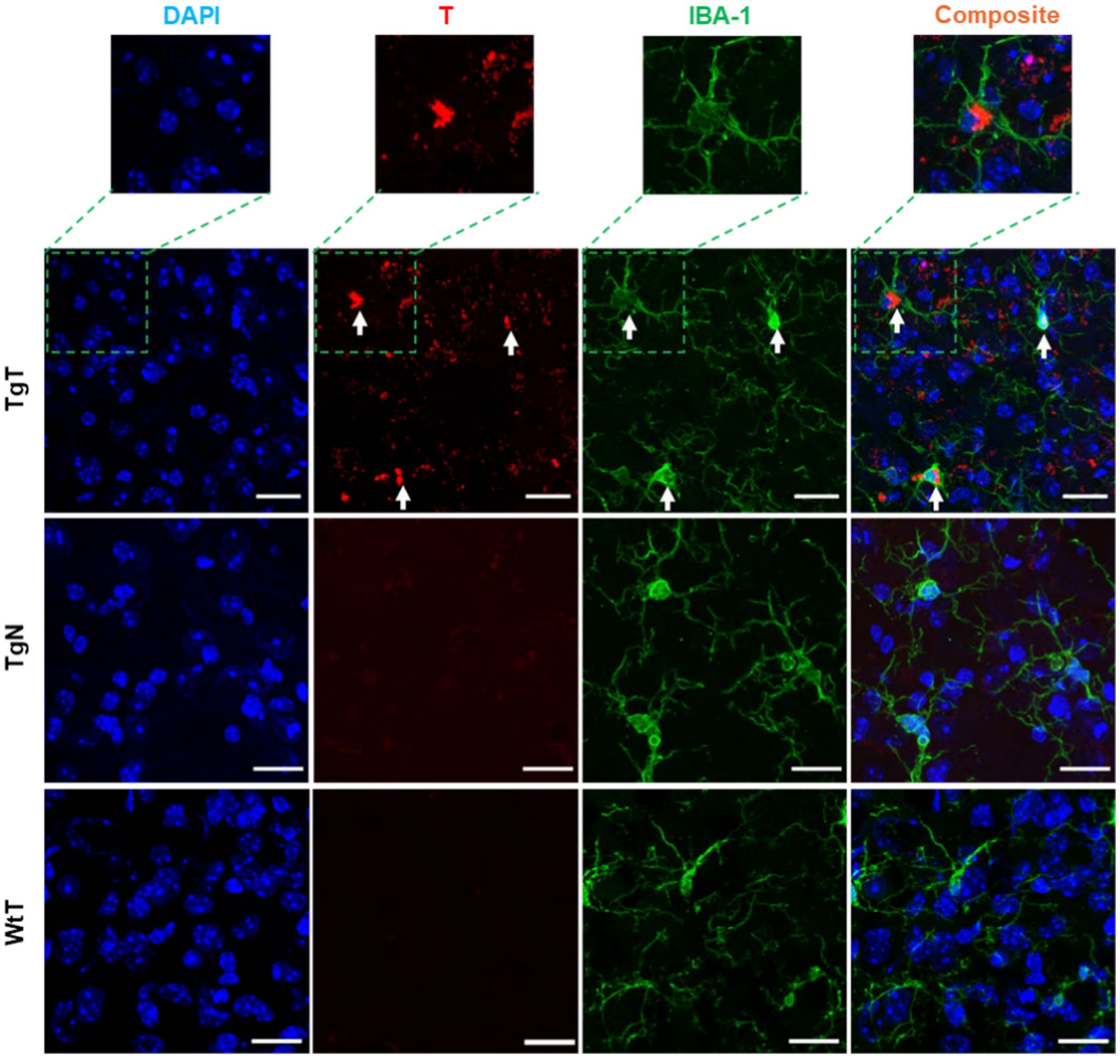
Microglia in the entorhinal cortex exhibits *in vivo* uptake of nano scavenger species with apparent compact structures. Brain tissue sections from 19-month-old TgT (*N* = 6) and TgN (*N* = 6) mice showing cell bodies and processes with strong IBA-1 reactivity in TgT sections also showed strong T signals (rhodamine, red), while TgN and WtT sections showed very faint to no T signal. Composite images created by merging signals from nuclear stain (DAPI), the T, and IBA-1 signals show that the strong compact T signal is within the cytosol of the IBA-1 reactive cell bodies. Scale bar = 20 μm.

IBA-1 is a well-established marker for microglia and macrophages and is upregulated during the activation of both cellular species. To assess the presence of microglia and their possible contribution to the observed *in vivo* MRI signal enhancement, brain tissue sections were stained to assess IBA-1 expression patterns. As exemplified by images from 19 month old mice (Figure 6), the stained tissue sections showed strong IBA-1 expression (green color) in both the TgT and TgN sections. However, the strong T signal in the TgT sections was absent in the TgN sections attributed to very little to no accumulation of the nontargeted particles, which is consistent with the low to no *in vivo* MRI signal enhancement in this cohort. A composite image created by merging the nuclei-stained image (DAPI), T, and IBA-1 fluorescence signals showed colocalization of IBA-1 reactivity with the compact structures with high rhodamine fluorescence intensity (white arrows). More importantly, the composite images also showed that the compact and highly fluorescent structures were cytosolic, suggesting *in vivo* engulfment of preformed T/oligomeric α-syn agglomerates by microglia. Images from other brain regions in the older cohorts as well as their 13 to 15-month-old counterparts (Supplementary Figures S8–S13) displayed similar rhodamine signal retention patterns and correlations between the rhodamine signal and IBA-1 reactivity. Taken together, these data strongly suggest that T encounters and forms agglomerates with oligomeric α-syn aggregates *in vivo* and that the resulting compact structures are engulfed by activated microglia, resulting in the accumulation of the contrast agent in this cell type, as hypothesized. However, the highly fluorescent structures are only a fraction of the total signal since signals from diffuse fluorescent structures do not appear to colocalize with IBA-1. To account for this signal and the role of the other cellular participants, contiguous tissue sections were stained for NF-H and glial fibrillary acidic protein (GFAP) reactivity to assess the roles of neurons and astrocytes, respectively.

### 3.8 Correlations between *in vivo* MR signal enhancement, nano scavenger signal, and NF-H immunoreactivity

**Figure 7:**
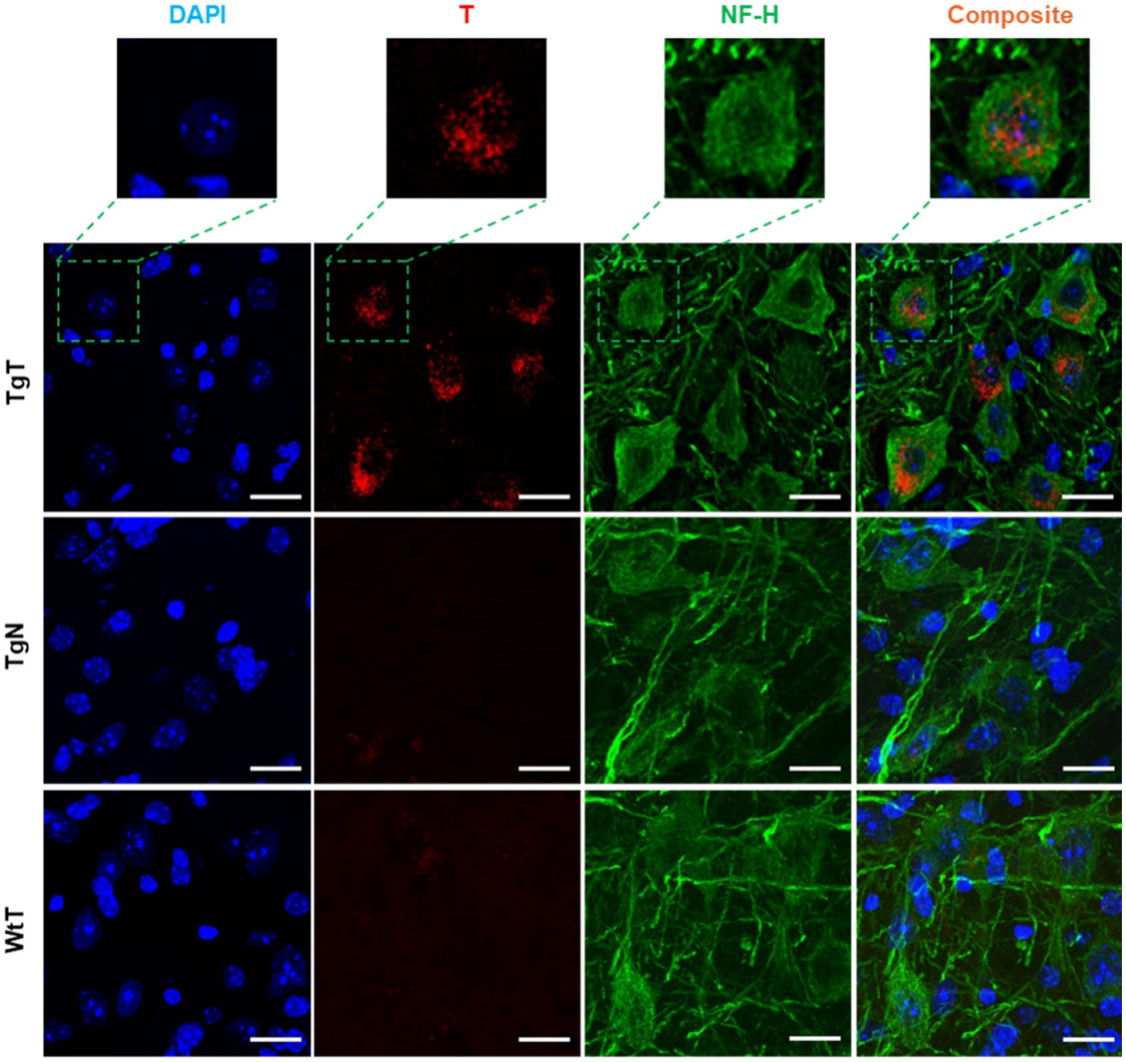
Neuronal cell bodies in the brainstem display *in vivo* uptake of nano scavenger species with apparent diffuse structural formations. Anti-NF-H-stained brain tissue displays T signal as well as cell bodies with strong anti-NF-H reactivity (green fluorescence, 750 nm). A composite image of both signals shows that the diffuse rhodamine signal is cytosolic. Scale bar = 20 μm.

NF-H is one of three intermediate filament proteins (including NF-L, NF-M and NF-H) found specifically in neurons. Antibodies against NF-H are useful for identifying neuronal cells and their processes in tissue sections and tissue culture. To assess the location of neurons relative to the T signal in the tissue and their contribution to the observed *in vivo* MRI signal enhancement, brain tissue sections were stained for anti-NF-H reactivity. Strong anti-NF-H reactivity (green color) was observed in tissue sections from all tested cohorts. Both cell bodies and axonal processes were also clearly visible. However, the cell bodies in the brain stem appeared to stain better than their counterparts in the cortical sections. The images also suggested that the NF-H reactive cell bodies correlated mostly with the diffuse T signal (red). As exemplified by images of stained tissue from the brain stem of 15-month-old mice (Figure 7), only the TgT cohorts showed significant rhodamine signal attributed to T, consistent with *in vivo* MR signal enhancement. A composite image generated by merging both signals with the nuclei stain (blue) shows that the diffuse T signal (yellow arrows) colocalizes with the anti-NF-H reactive cell bodies. Furthermore, the signal is cytosolic, suggesting *in vivo* uptake of T-labeled α-syn oligomers by neurons. Images from other brain regions as well as the other age groups (Supplementary Figures S14, S15) showed similar patterns.

### 3.9 Correlations between *in vivo* MRI signal enhancement, nano scavenger signal, and GFAP immunoreactivity

**Figure 8:**
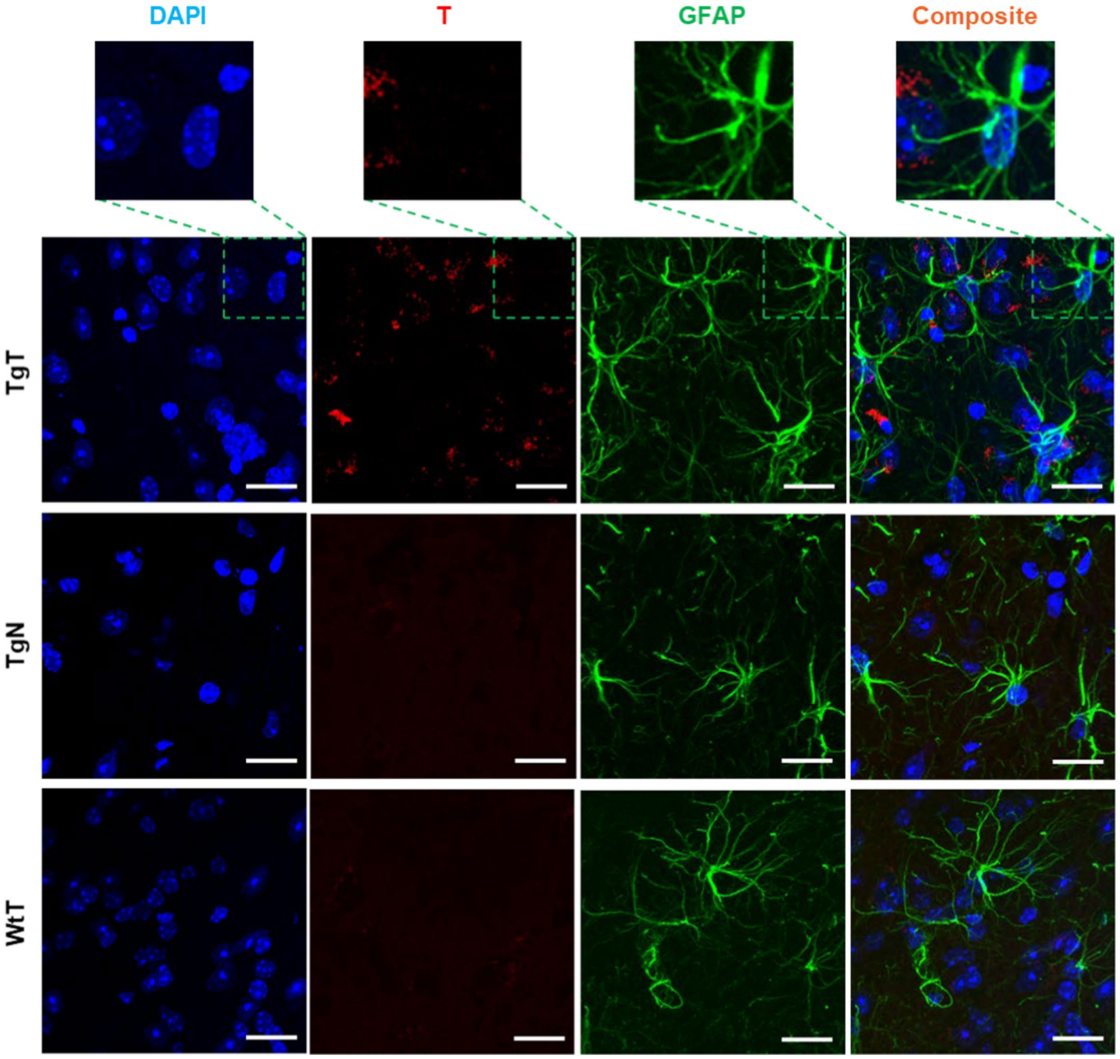
Anti-GFAP-stained sections of the entorhinal cortex show T signal as well as strong GFAP reactivity for TgT mice against controls, but composite images do not show conclusive evidence of the internalization of T by GFAP-reactive cells. Scale bar = 20 μm.

GFAP is an intermediate filament protein principally found in astrocytes in the CNS, but it can also be found in neurons, hepatic stellate cells, kidney mesangial cells, pancreatic stellate cells, and Leydig cells. GFAP acts as an intracellular structural component of the astrocytic cytoskeleton, and the expression of the GFAP gene has attracted considerable attention because its onset is a marker of astrocyte development, and its upregulation is a marker of reactive astrogliosis. To understand T and astrocyte interactions *in vivo* and their relationship to the observed MR signal enhancement, brain sections were immunoassayed with a GFAP antibody. Images of stained tissue (Figure 8) showed strong anti-GFAP reactivity with the characteristic asterisk structures of reactive astroglia in all brain sections of the TgT and TgN mice. This reactivity cooccurred with the T signal (rhodamine, red fluorescence), suggesting a correlation between astrocyte reactivity and the observed *in vivo* MRI signal enhancement. However, composite images generated by merging the T and GFAP signals with the nuclei-stained image (DAPI) did not show unequivocal cytosolic colocalization of the T signal in astrocytes, as observed in microglia and neurons in any brain region. Images from other brain regions and age groups are shown in Supplementary Figures S16–S21.

## 4 Discussion

The data from this study strongly supports our nano scavenger/oligomeric α-syn/reactive gliosis/neuronal interplay hypothesis. Our *in vitro* data demonstrated that exposing T to α-syn fibrils resulted in the formation of agglomerates. The DLS data show agglomerate species with hydrodynamic diameters ranging from 200 nm to >1 micrometer at fibril concentrations of 0.1 μg/mL. This particle size range was corroborated by TEM measurements, which revealed different T/fibril species within the same size range. Some reports in the literature suggest that the CSF concentration of oligomeric α-syn in PD patients can be as high as 170 ng/mL (Tokuda et al., 2010; Majbour et al., 2016). The lack of agglomerates when N was exposed to fibrils at concentrations up to 0.5 μg/mL, the reduction in agglomerate size and no agglomerates in the saturated free ligand blocking experiments all demonstrated that agglomerate formation results from the specific binding of ligands on the surface of T to ligand binding sites on the fibrils.

The data from our *in vitro* cell uptake studies equally support this hypothesis. Particles in the brain >0.5 microns in diameter are particularly suited for phagocytosis, and microglia are the principal modulators of phagocytosis in the CNS (Flannagan et al., 2012; Galloway et al., 2019). While both microglial and neuronal cell lines showed avid uptake of fibrils under all tested conditions, there was little evidence of uptake by the tested astrocyte cell line. More importantly, although the neuroblastoma and microglial cell lines internalized α-syn fibrils within 1.5 h of exposure, T alone, was not internalized within the same time frame except in the presence of fibrils, while the N formulation, which lacks targeting ligands, was not internalized in the presence of fibrils within the same time frame. These findings align with the assertion that oligomeric α-syn aids in the internalization and accumulation of T in cells.

More importantly, our *in vitro* data appears to translated seamlessly into our *in vivo* results. As hypothesized, noninvasive *in vivo* brain MR images showed statistically significant signal enhancement in TgT mice compared to controls, attributed to the accumulation of the contrast agent. Immunohistochemical analysis of the treated brain tissue revealed a strong correlation between the regional distribution of the *in vivo* signal and Lewy pathology. Although there was no independent confirmation of the presence of oligomeric α-syn species in the tissue, we assumed that the intracellular Lewy bodies and neurites were in flux with oligomeric species in the CSF and IF. IBA-1 reactivity revealed correlation of microglia activity with the MR signal and cytosolic inclusions with strong T fluorescent signals were clearly visible within their cell bodies. We speculate that this strong signal comes from larger preformed T/oligomeric α-syn agglomerate species engulfed by microglia via phagocytosis. Some reports in the literature suggest that microglia can engulf oligomeric α-syn into autophagosomes for degradation (Choi et al., 2020), and one can expect that if fluorescently labeled, α-syn oligomers in autophagosomes would also appear as bright structures within microglial cell bodies. Indeed, we believe that this is what is observed in the confocal microscopy images of HMC-3 cells exposed to labeled α-syn fibrils (Figure 3). On the other hand, endogenous α-syn is not fluorescently labeled, and the red fluorescence in the microglia in brain tissue sections of TgT mice can only originate from T. Taken together with our *in vitro*, as well as our *in vivo* control data showing no signal enhancement in TgN mice, the cytosolic red fluorescence in the microglia is likely due to internalization of larger preformed T/oligomeric α-syn complex species in the CSF and IF by the cells. Similarly, the diffuse cytosolic red fluorescence observed in the anti-NF-H-labeled cell bodies is attributed to the internalization of smaller preformed T/oligomeric α-syn species by neurons. This would most likely occur via similar mechanisms as conventional endocytosis or receptor-mediated internalization of native oligomeric α-syn by neurons (Mavroeidi and Xilouri, 2021). Surprisingly, despite several reports on astrocyte involvement in the spread of α-syn pathology (Lee et al., 2010; Mavroeidi and Xilouri, 2021), we found no clear evidence of avid internalization of fibrils (with or without T) by T98G cells *in vitro* or T signals in GFAP-expressing cells *in vivo*. However, these results are consistent with reports that microglia are the principal scavengers of extracellular oligomeric α-syn (Zhang et al., 2018; Mavroeidi and Xilouri, 2021).

## 5 Conclusion

This pilot study paves the way for a novel approach to simultaneously profile α-syn accumulation and microgliosis using *in vivo* molecular MRI in the M83 α-synuclein mouse model after a single injection. The *in vitro* DLS and TEM data demonstrated that a spectrum of agglomerate species with sizes ranging from 200 nm to >1 μm in diameter are generated upon exposure of T to α-syn fibrils *in vitro*. The *in vitro* cell uptake studies showed avid internalization of fibrils with or without T, but avid and rapid uptake of T was only possible in the presence of fibrils. The tested astrocyte cell line did not display unequivocal internalization of either fibrils or T when used separately or in combination. *In vivo* MRI demonstrated that transgenic mice injected intravenously with the agent showed statistically significant brain signal enhancement due to retention of the agent, while *ex vivo* immunohistochemical analysis demonstrated that microglia and neurons were strongly involved in retention of the agent in the brain. In addition, *in vivo* brain MRI signal enhancement showed correlation with both the accumulation of Lewy pathology and microglial activity. To the best of our knowledge, no other report has demonstrated this type of interplay between nanoparticles and oligomeric α-syn or any of the other amyloid aggregates with microglia and neurons. Although the fate of the internalized agglomerates in this study was not determined, a recent report demonstrated that microglia engulf α-syn aggregates into autophagosomes where they are digested. Our data demonstrating *in vitro* and *in vivo* internalization of T are overwhelming, but we note that the uptake mechanism is rather speculative; the fate of the internalized agent is not yet understood; and mice with very early stage disease, which can offer greater insight into the disease’s early development and propagation, were not tested. We are currently investigating each of these areas and will report our findings in due course.

## Data availability statement

The datasets presented in this study can be found in online repositories. The names of the repository/repositories and accession number(s) can be found in the article/Supplementary material.

## Ethics statement

Ethical approval was not required for the studies on humans in accordance with the local legislation and institutional requirements because only commercially available established cell lines were used. The animal study was approved by the Institutional Animal Care and Use Committee. The study was conducted in accordance with the local legislation and institutional requirements.

## Author contributions

XS: Writing – review & editing, Writing – original draft, Methodology, Formal analysis, Data curation. AB: Writing – review & editing, Methodology, Formal analysis, Data curation. PB: Writing – review & editing, Methodology. JC: Writing – review & editing. AA: Writing – review & editing, Resources. ET: Writing – review & editing, Writing – original draft, Resources, Formal analysis, Data curation.

## Glossary

α-syn: Alpha-synuclein
AD: Alzheimer’s disease
Aβ: Amyloid-β
NF-H: Anti-neurofilament H
IBA-1: Anti-ionized calcium-binding adaptor molecule 1
BBB: Blood-brain barrier
CSF: Cerebrospinal fluid
DAB staining: 3,3′-Diaminobenzidine staining
DLS: Dynamic light scattering
FSE-IR: Fast spin-echo inversion recovery
HBS: Histidine-buffered saline
ICP-AES: Inductively coupled plasma-atomic emission spectrometry
ICP-OES: Inductively coupled plasma-optical emission spectrometry
IVC: Inferior vena cava
IACUC: Institutional Animal Care and Use Committee
ISF: Interstitial fluid
MRI: Magnetic resonance imaging
N: Nontargeted control formulation
PD: Parkinson’s disease
PET: Positron emission tomography
ROIs: Regions of interest
SPECT: Single-photon emission computed tomography
T: α-syn-targeted formulation
TgN: Transgenic mice treated with N
TgT: Transgenic mice treated with T
TLR-4: Toll-like receptor 4
WtT: Wild-type age-matched cohorts treated with T
