# Supplementary material for: A dual target molecular magnetic resonance imaging probe for noninvasive profiling of pathologic alpha-synuclein and microgliosis in a mouse model of Parkinson’s disease
Source: Front Neurosci. 2024 Jul 24;18:1428736. doi: 10.3389/fnins.2024.1428736 (PMC11303179; doi:10.3389/fnins.2024.1428736)
Supplement: Supplementary file 1 [file Data_Sheet_1.PDF]

**Supplementary Information**

# A dual target molecular MRI probe for noninvasive profiling of pathologic alpha-synuclein and microgliosis in a mouse model of Parkinson's disease.

Xianwei Sun,[a] Andrew Badachhape,[a] Prajwal Bhandari,[a] Jeannie Chin,[b] Ananth Annapragada,[a,c] Eric Tanifum.[a,c*]

[a]Department of Radiology, Baylor College of Medicine, Houston, TX 77030, USA

[b]Department of Neuroscience, Baylor College of Medicine, Houston, TX 77030, USA

[c]Department of Radiology, Texas Children's Hospital, Houston, TX 77030, USA

*Corresponding author: tanifum@bcm.edu

## TABLE OF CONTENTS

1. Experimental methods and procedures
2. Supplementary figures.
3. NMR and MALDI spectra.

## General information and methods

**Materials**. Unless otherwise noted, all reagents and solvents were obtained from commercial sources including, Sigma-Aldrich, TCI, and Acros Organics and used without further purification. Thin layer chromatography (TLC) was performed on silica gel 60 F254 plates from EMD Chemical Inc. and components were visualized by ultraviolet light (254 nm and ) and/or phosphomolybdic acid, 20 wt% solution in ethanol. SiliFlash silica gel (230–400 mesh) was used for all column chromatography.

**Measurements**. Proton nuclear magnetic resonances ($^1$H NMR) were recorded at 600 MHz or 500 MHz on Bruker 600 or 500 NMR spectrometers. Carbon nuclear magnetic resonances ($^{13}$C NMR) were recorded at 75 MHz or 125 MHz on a Bruker 300 or 500 NMR spectrometers respectively. Chemical shifts are reported in parts per million (ppm) from an internal standard chloroform (7.26 ppm), or dimethylsulfoxide (2.50 ppm) for $^1$H NMR; and from an internal standard of either residual chloroform (77.00 ppm), or dimethylsulfoxide (39.52 ppm) for $^{13}$C NMR. NMR peak multiplicities are denoted as follows: s (singlet), d (doublet), t (triplet), q (quartet), dd (doublet of doublet), td (doublet of triplet), dt (triplet of doublet), and m (multiplet). Coupling constants (*J*) are given in hertz (Hz). High resolution mass spectra (HRMS) were obtained from The Ohio State University Mass Spectrometry and Proteomics Facility. The mean particle size of liposomes was determined using a Dynamic Light Scattering (DLS) instrument (Brookhaven Instruments Corp., Holtsville, NY, USA). Tissue and formulation elemental analysis was determine using Inductively coupled plasma mass spectrometry (ICP-MS). Negative-stain Transmission Electron Microscopy (TEM) images were captured from a JEOL 3200FSC (300kV) electron microscope. Magnetic Resonance Imaging (MRI) was performed on a 1 T permanent magnet scanner (M2 system, Aspect Imaging, Shoham, Israel). All confocal microscopy images were captured from an Olympus IX81 microscope, and the images were processed and analyzed using Fiji-ImageJ software.

***Chemical synthesis***.

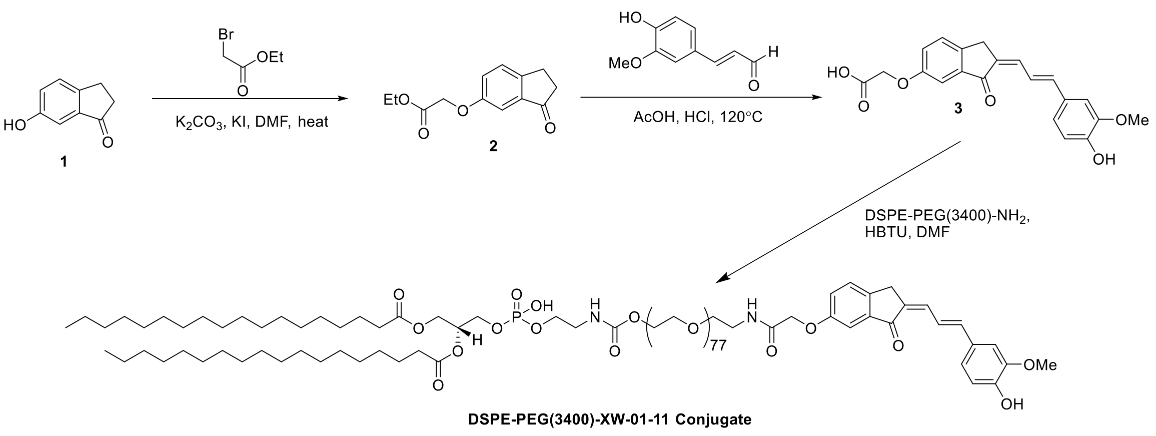


***Scheme S1***. *Synthetic route to DSPE-PEG(3400)-XW-01-11 Conjugate.*

**Ethyl 2-((3-oxo-2,3-dihydro-1H-inden-5-yl)oxy)acetate (2)**: Ethyl bromoacetate (2.3 g, 13.5 mmol) was added in one portion to a solution of 6-hydroxy-1-indanone, **1** (1.0 g, 6.8 mmol), $K_2CO_3$ (2.8 g, 20.2 mmol) and KI (112 mg, 0.7 mmol) in DMF (10 mL). The reaction mixture was stirred at 90 °C overnight. At this point, TLC (silica, 1:3 EtOAc–hexanes) showed that the reaction was complete. The reaction mixture was cooled to room temperature, and then filtered through a pad of celite using ethyl acetate (50 mL) as the eluent and the filtrate concentrated. The residue was purified by column chromatography eluted with ethyl acetate/hexanes gradient to afford the desired product **2** (1.4 g, 90%) as a white solid. $^1$H NMR (600 MHz, $CDCl_3$) δ 7.38 (d, $J$ = 8.4 Hz, 1H), 7.26 (dd, $J_1$ = 2.4 Hz, $J_2$ = 8.4 Hz, 1H), 7.10 (d, $J$ = 2.4 Hz, 1H), 4.64 (s, 2H), 4.26 (q, $J$ = 7.8 Hz, 2H), 3.06 (t, $J$ = 6.0 Hz, 2H), 2.69 (t, $J$ = 6.0 Hz, 2H), 1.29 (t, $J$ = 7.8 Hz, 3H); $^{13}$C NMR (150 MHz, $CDCl_3$) δ 206.6, 168.3, 157.5, 148.8, 138.1, 127.6, 124.3, 105.7, 65.4, 61.4, 36.8, 25.0, 14.0.

**2-{[(Z)-2-((E)-3-(4-Hydroxy-3-methoxyphenyl)allylidene]-3-oxo-2,3-dihydro-1H-inden-5-yl)oxy}acetic acid** (**3**): A solution of 4-hydroxy-3-methoxy-cinnamaldehyde (588 mg, 3.3 mmol) and compound **2** (700 mg, 3.0 mmol) in acetic acid (10 mL) was slowly added 37% HCl (0.2 mL). The reaction mixture was stirred at 120 °C overnight, and then cooled to room temperature. The cooled solvent was poured into ice water and then filtered out. The solid was recrystallized with methanol to afford the desired product **3** (768 mg, 70%) as a brown solid. $^1$H NMR (600 MHz,

DMSO-$d_6$) δ 13.09 (s, 1H), 9.52 (s, 1H), 7.55 (d, *J* = 8.4 Hz, 1H), 7.32-7.24 (m, 3H), 7.13 (dd, $J_1$ = 6.0 Hz, $J_2$ = 9.0 Hz, 1H), 7.08 (d, *J* = 5.4 Hz, 1H), 7.06 (td, $J_1$ = 3.0 Hz, $J_2$ = 7.8 Hz, 1H), 6.81 (d, *J* = 8.4 Hz, 1H), 4.79 (s, 2H), 3.85 (s, 3H), 3.84 (s, 2H); $^{13}$C NMR (150 MHz, DMSO-$d_6$) δ 192.2, 170.1, 157.6, 148.5, 147.9, 142.9, 142.1, 139.9, 135.3, 133.7, 127.9, 127.5, 123.2, 122.1, 121.9, 115.7, 110.5, 106.3, 64.8, 55.7, 29.5.

**DSPE-PEG(3400)- XW-01-11 Conjugate**: A solution of DSPE-PEG(3400)-$NH_2$ (500 mg, 0.1 mmol) and compound **3** (120 mg, 0.3 mmol) in dry DMF (8 mL) was added HSTU (160 mg, 0.4 mmol). The reaction mixture was stirred at room temperature two days overnight. The reaction mixture was concentrated under reduced pressure.   The residue was diluted with methanol/water mixture (1:1, 8 ml), and then loaded into a 2000 MWCO dialysis bag and dialyzed against MES buffer (50mM, 2×5 liters) for 8 hours and then water (3×5 liters) for 2 days. The water was then removed by freeze drying to obtain the desired compound as a yellow solid (242 mg, 48%)  . $^1$H NMR (600 MHz, $CDCl_3$) δ 7.45 (d, *J* = 8.4 Hz, 1H), 7.23 (dd, $J_1$ = 2.4 Hz, $J_2$ = 5.6 Hz, 1H), 7.19-7.17 (m, 2H), 7.15 (d, *J* = 2.4 Hz, 1H), 7.02–6.99 (m, 3H), 6.78 (dd, $J_1$ = 8.4 Hz, $J_2$ = 10.8 Hz, 1H), 5.09 (s, 1H), 4.48 (s, 2H), 4.30 (dd, $J_1$ = 2.4 Hz, $J_2$ = 12.0 Hz, 1H), 4.12–4.06 (m, 2H), 4.01 (t, *J* = 4.2 Hz, 2H), 3.98 (s, 2H), 3.81–3.77 (m, 5H), 3.73 (s, 2H), 3.49 (dd, $J_1$ = 2.4 Hz, $J_2$ = 7.8 Hz, 2H), 3.48–3.46 (m, 3H), 3.35–3.32 (m, 4H), 3.20–3.18 (m, 2H), 2.21–2.18 (m, 4H), 1.93–1.92 (m, 4H), 1.50–1.47 (m, 4H), 0.761 (t, *J* = 6.0 Hz, 6H); HRMS (MALDI) *calcd* for $C_{219}H_{410}N_2O_{92}P$ $[M+H]^+$ 4571.7203, found, 4571.7069.

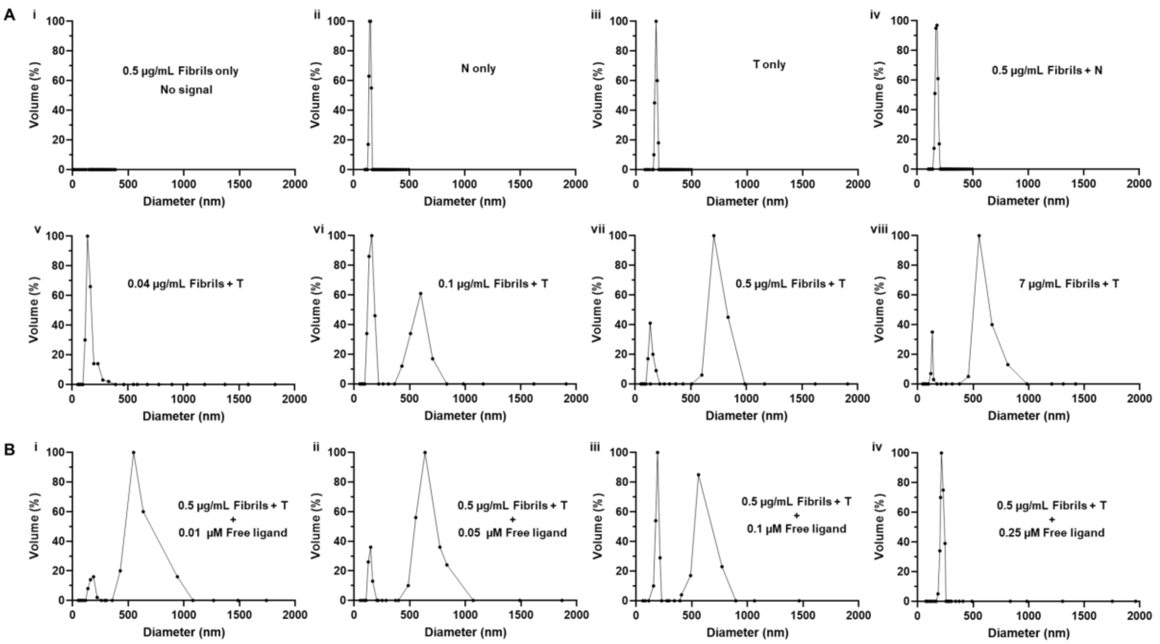


**Figure S1**. *In vitro* characterization of **T**/α-syn fibril agglomerate formation by DLS. All DLS experiments were run at the same lipid concentration (10 µM lipid) for the **T** and **N** formulations. (**A**) DLS profiles of solutions of fibrils at different concentrations with the liposome formulations: (**A.i**) A 0.5 µg/mL solution of synthetic α-fibrils show no DLS signal; (**A.ii**) A solution of **N** shows particle distribution of ~150 nm hydrodynamic diameter; (**A.iii**) A solution of **T** shows particle distribution of ~185 nm hydrodynamic diameter; (**A.iv**) A 0.5 µg/mL solution of synthetic α-syn fibrils incubated with **N** shows no major change particle distribution; (**A.v**) A 0.04 µg/mL solution of synthetic α-fibrils incubated **T** shows slight distortion at the base of the particle distribution peak; (**A.vi**) Increase in fibrils concentration to 0.1 µg/mL results in two different particle populations, the original particle population with a hydrodynamic diameter at ~185 nm and a new population with a hydrodynamic diameter of ~600 nm, attributed to **T**/fibril agglomerate formation; (**A.vii**) A 0.5 µg/mL solution of synthetic α-fibrils incubated **T** shows two different particle populations, the major particle population with a hydrodynamic diameter of ~750 nm; (**A.viii**) Increase to 7 µg/mL fibrils incubated with **T** shows more decrease in the small particle size distribution. (**B)** The blocking experiment was run at the same lipid concentration (10 µM lipid) and α-syn fibrils concentration (0.5 µg/mL), DLS profiles of solutions of free ligand (**XW-01-11**) at different concentrations with the liposome formulations and α-syn fibrils: **(B.i**) The free ligand at 0.01 µM concentration shows no major change in the particle distribution; **(B.ii**) Increase of the free ligand concentration to 0.05 µM shows an increase in the small particle population suggesting partial blocking of binding site on fibrils; **(B.iii**) Further increase of the free ligand concentration to 0.1 µM suggests increase blocking of binding sites on fibrils by the free ligand; (**B.iv**) Increase in free ligand concentration to 0.25 µM shows nor large particles form, suggesting complete saturation of binding sites on fibrils.

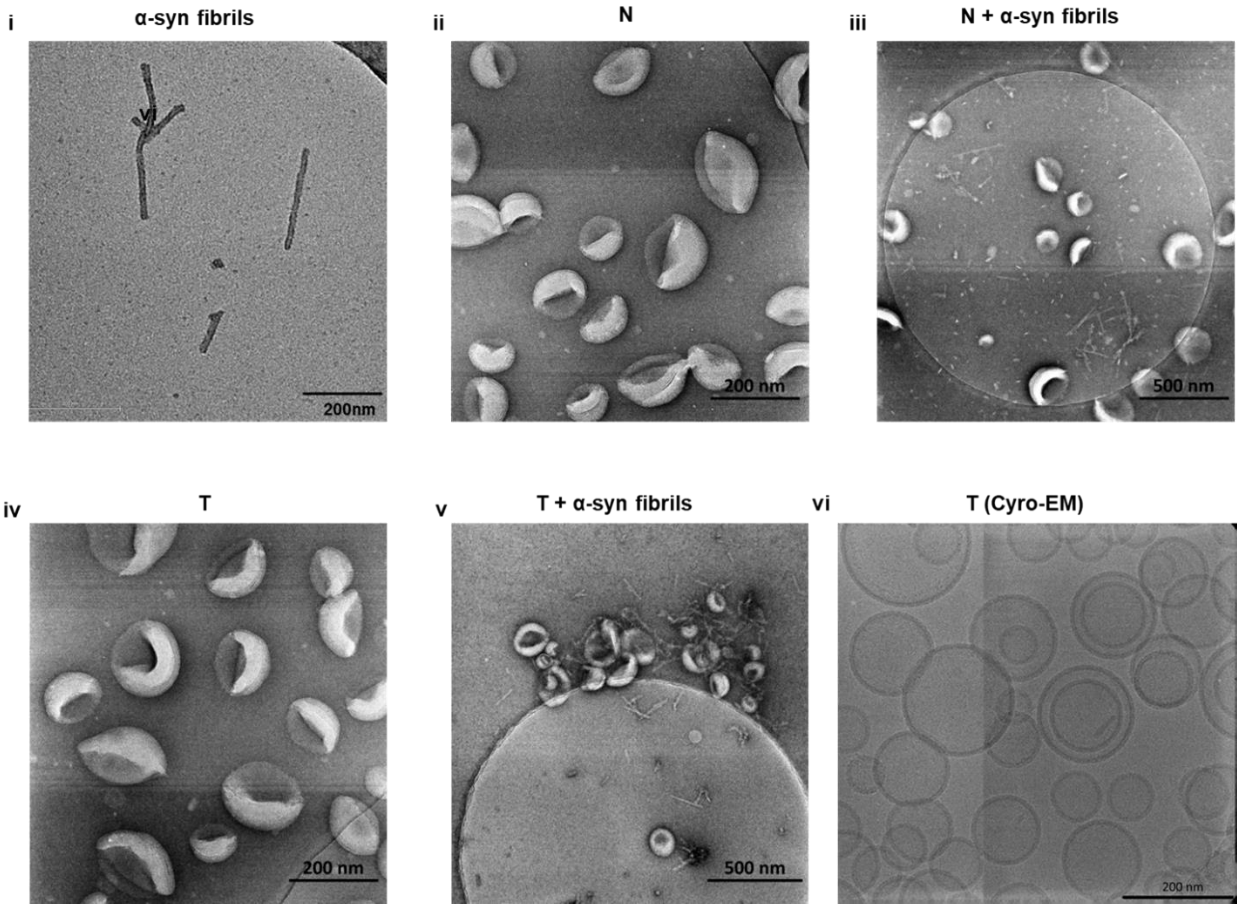


**Figure S2**. TEM images of α-syn fibrils exposed to **T** or **N**: (**i**) TEM image of α-syn fibrils; (**ii**) TEM image of **N**; (**iii**) TEM image of **N** incubated with α-synuclein fibrils; (**iv**) TEM image of **T**; (**v**) TEM image of a nanometer size cluster of **T** bound to fibrils; (**vi**) Cyro-EM image of **T**.

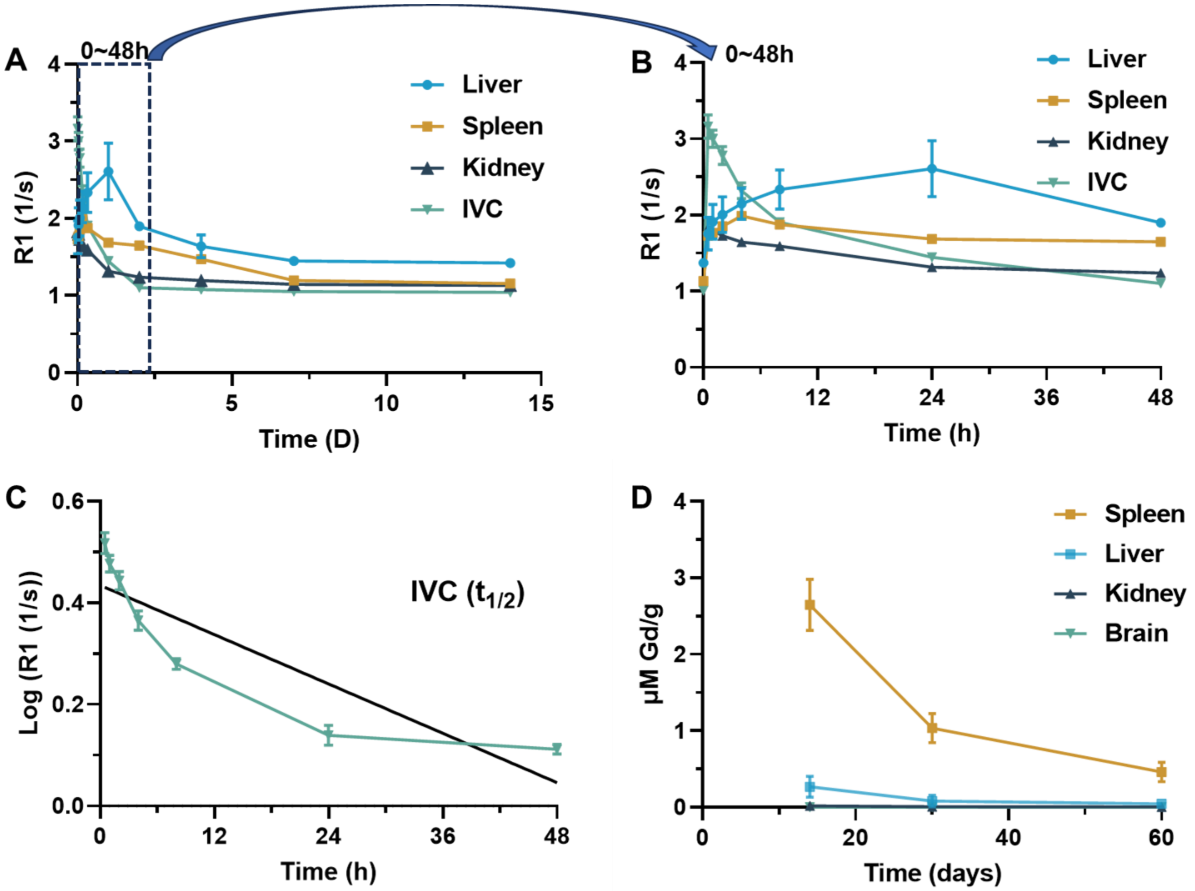


**Figure S3.** Pharmacokinetics and organ distribution. *In vivo* MRI studies were performed in C57BL/6 mice (n=3, 9-12 weeks old) on a 1T permanent magnet (M2 system, Aspect Imaging, Shoham, Israel) with a 35 mm transmit-receive RF volume coil. (**A**) Plot of MRI signal in blood (inferior vena cava) and select organs (liver, spleen, and kidneys) against time over 14 days following contrast injection; (**B**) Plot of MRI signal against time between 0 h to 48 hours; (**C**) Determination of half-life ($t_{1/2}$); (**D**) Distribution of Gd(III) in select organs (liver, spleen, brain, kidney) from 14 days to 60 days determined by tissue analysis using ICP.

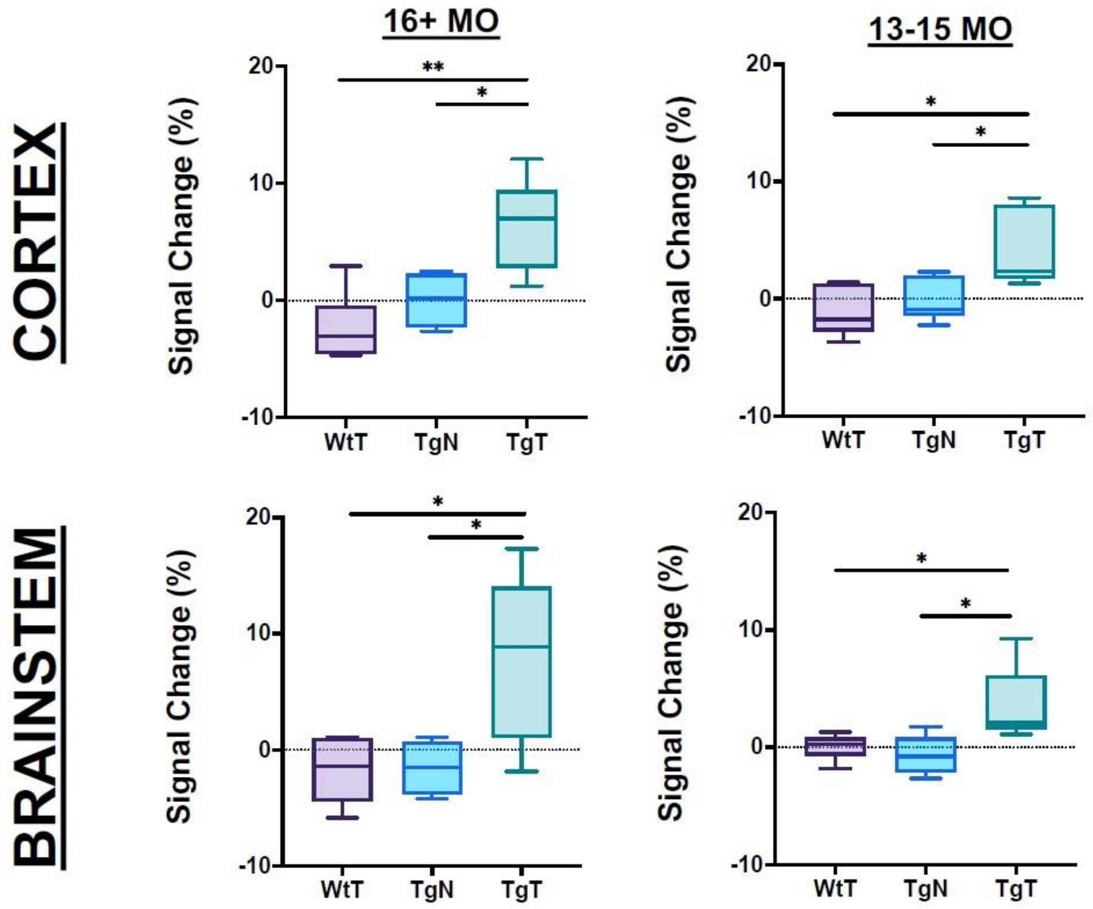


**Figure S4**. Box and whisker plots of percent signal enhancement in the cortex and brainstem of all age groups tested show statistically significant mean enhancements in **TgT** against **TgN** and **WtT**. Statistics: Kruskal–Wallis (Pairwise – Bonferroni: * $p<0.05$, ** $p<0.005$.

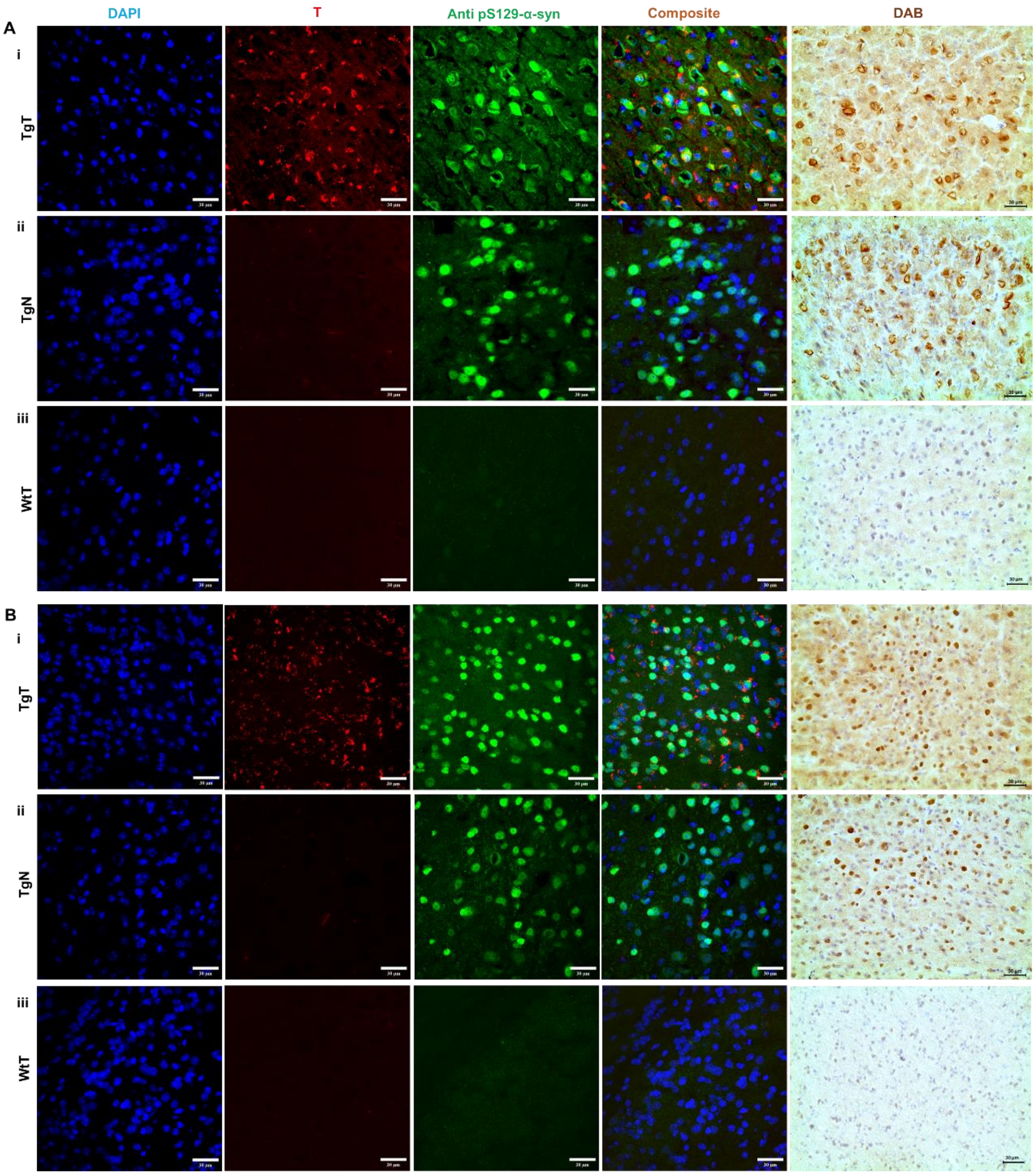


**Figure S5.** Immunohistochemical analysis demonstrates a correlation between *in vivo* MRI signal enhancement, nano scavenger location and Lewy pathology in the olfactory bulb. (**A**) Patterns of anti-pSer129-α-syn reactivity observed in sections from a 16 to 19-month-old mice: (**A.i**) **TgT** mouse shows colocalization of **T** (rhodamine fluorescence) with anti pS129-α-syn antibody signal (Lewy pathology). Lewy pathology is confirmed with DAB staining; (**A.ii**) **TgN** mice shows little to no rhodamine fluorescence signal (consistent with *in vivo* MRI), but strong pS129-α-syn pathologic structures (transgenic mice); (**A.iii**) **WtT** mice show no rhodamine fluorescence signal and no anti-pS129-α-syn activity; (**B**) Anti-pSer129-α-syn stained olfactory bulb tissue sections from 13 to 15-month-old mice display similar Lewy pathology patterns, albeit less dense. Scale bar = 30 µm

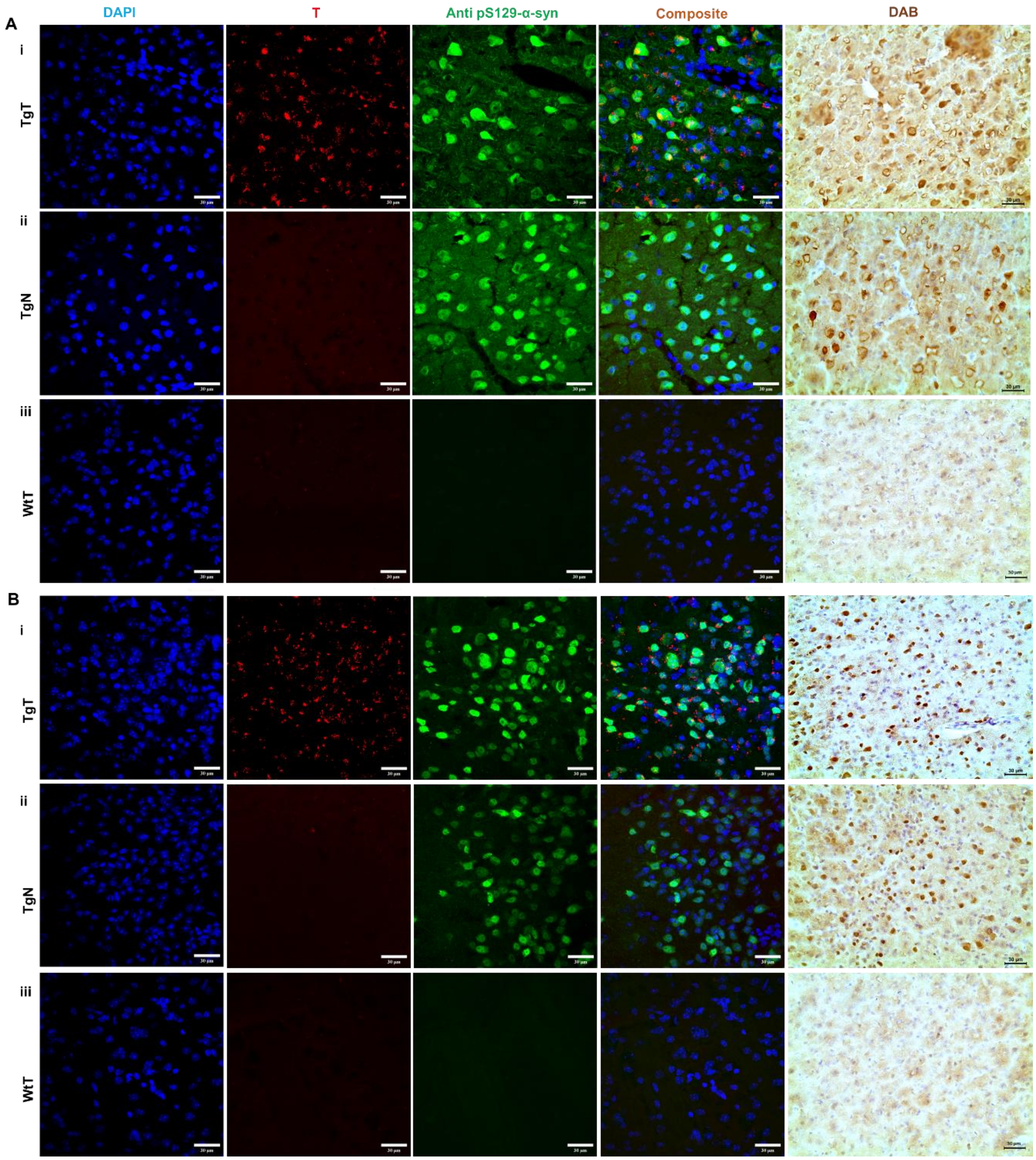


**Figure S6.** Immunohistochemical analysis demonstrates a correlation between *in vivo* MRI signal enhancement, nano scavenger location and Lewy pathology in the entorhinal cortex. (**A**) Patterns of anti-pSer129-α-syn reactivity observed in stained tissue sections from a 16 to 19-month-old mice: (**A.i**) **TgT** mice show colocalization of **T** (rhodamine fluorescence) with anti-pS129-α-syn antibody signal (Lewy pathology). Lewy pathology is confirmed with DAB staining; (**A.ii**) **TgN** mice show little to no rhodamine fluorescence (consistent with *in vivo* MRI), but strong pS129-α-syn pathologic structures (transgenic mice); (**A.iii**) **WtT** mice show no rhodamine fluorescence and no anti-pS129-α-syn reactivity; (**B**) Tissue sections from 13 to 15-month-old mice show similar patterns, albeit less dense. Scale bar = 30 µm.

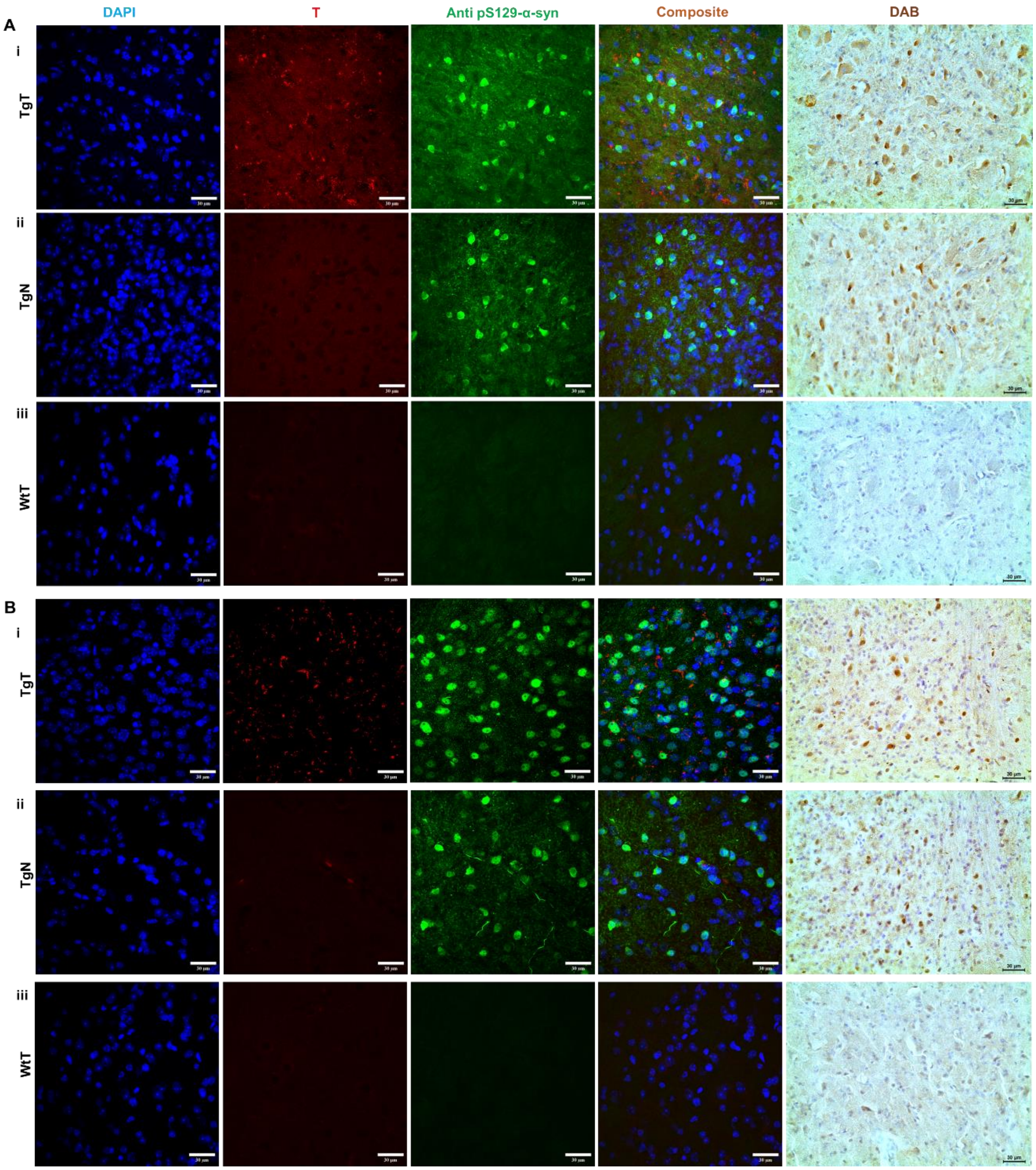


**Figure S7.** Immunohistochemical analysis demonstrates a correlation between *in vivo* MRI signal enhancement, nano scavenger location, and Lewy pathology in the brain stem. (**A**) Patterns observed in anti-pSer129-α-syn stained tissue sections from 16 to 19-month-old mice: (**A.i**) **TgT** mice show colocalization of **T** (rhodamine fluorescence) with anti-pS129-α-syn antibody signal (Lewy pathology). Lewy pathology is confirmed with DAB staining; (**A.ii**) **TgN** mice show little to no rhodamine fluorescence (consistent with *in vivo* MRI), but strong pS129-α-syn pathologic structures (transgenic mice); (**A.iii**) **WtT** mice show no rhodamine fluorescence and no anti-pS129-α-syn activity; (**B**) Anti-pSer129-α-syn stained brainstem tissue sections from a 13 to 15-month-old mice similar patterns of Lewy pathology patterns, albeit less dense. Scale bar = 30 µm.

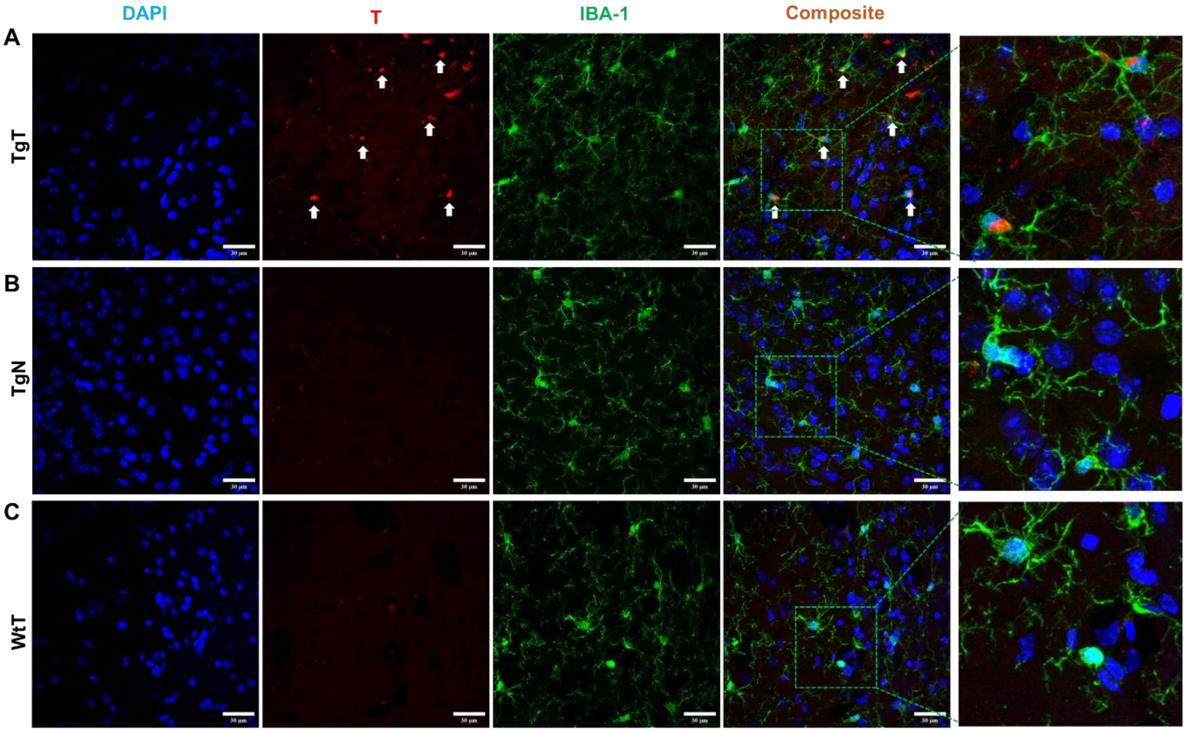


**Figure S8**. Microglia display *in vivo* uptake of nano scavenger species with apparent compact structural formation (white arrows) in the olfactory bulb as 16 to19-month-old **TgT**, **TgN** and **WtT** mice all show cell bodies and processes with strong IBA-1 reactivity (**A**) Composite images from imaged **TgT** tissue sections show colocalization of **T** signal (rhodamine) in cell bodies of anti-IBA-1 reactive cells; Control sections, **TgN** and **WtT** (**B** and **C** (respectively) show very faint to no signal in the rhodamine channel. Scale bar = 30 µm.

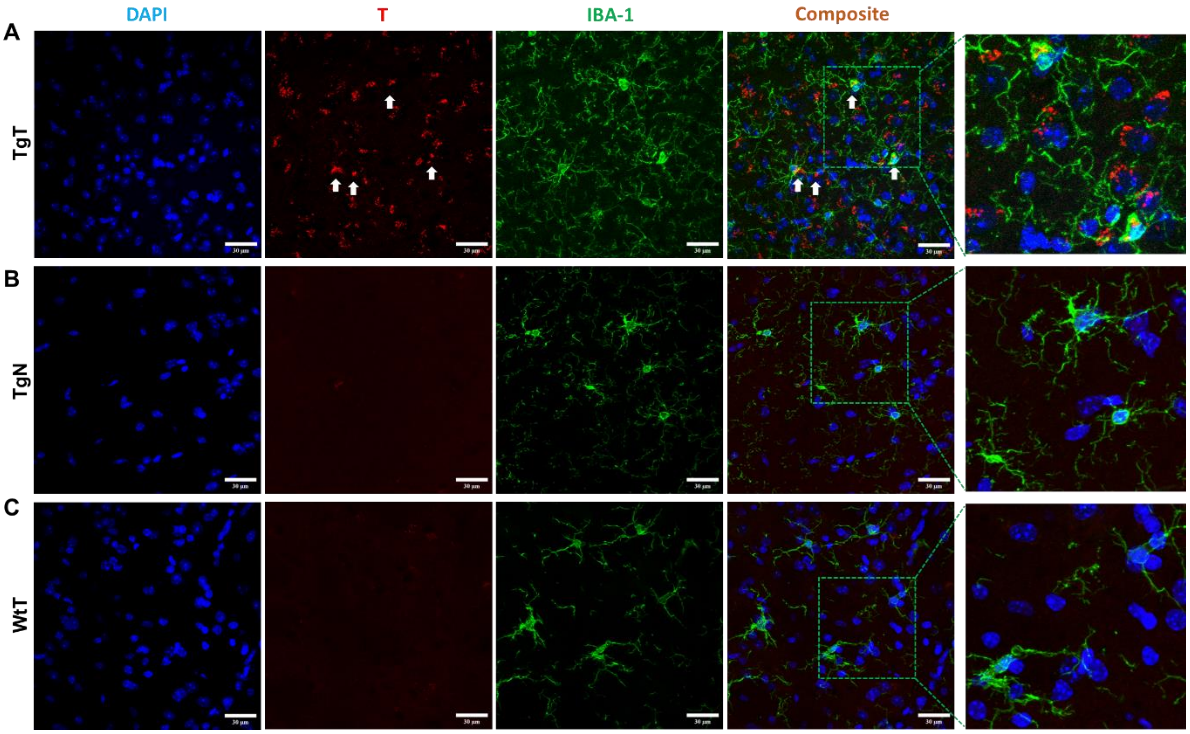


**Figure S9**. Microglia display *in vivo* uptake of nano scavenger species with apparent compact structural formation (white arrows) in the olfactory bulb as 13 to 15-month-old **TgT**, **TgN** and **WtT** mice all show cell bodies and processes with strong IBA-1 reactivity (**A**) Composite images from imaged **TgT** tissue sections colocalization of NS signal (Rhodamine) in cell bodies of anti-IBA-1 reactive cells; Control sections, **TgN** and **WtT** (**B** and **C** respectively) show very faint to no signal in the rhodamine channel. Scale bar = 30 µm.

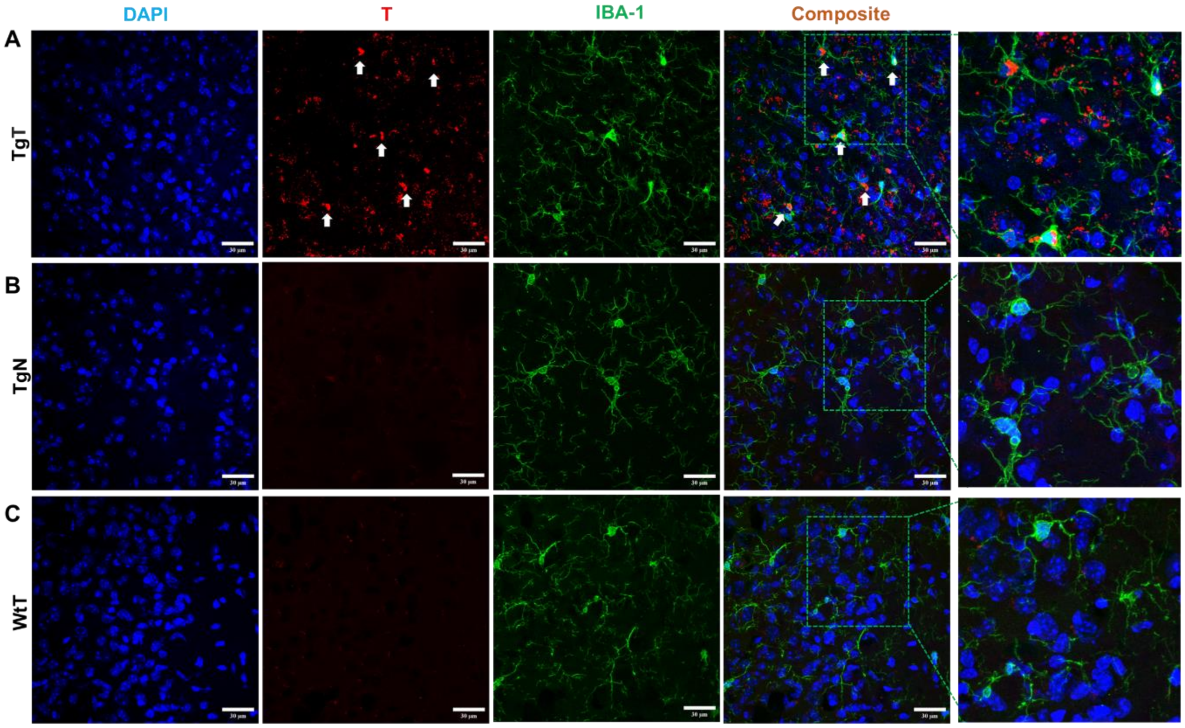


**Figure S10**. Microglia display *in vivo* uptake of nano scavenger species with apparent compact structural formation (white arrows) in the entorhinal cortex as 16 to19-month-old **TgT**, **TgN** and **WtT** mice all show cell bodies and processes with strong anti-IBA-1 reactivity (**A**) Composite images from imaged **TgT** tissue sections show colocalization of **T** signal (rhodamine) in cell bodies of anti-IBA-1 reactive cells; Control sections, **TgN** and **WtT** (**B** and **C** respectively) show very faint to no signal in the rhodamine channel. Scale bar = 30 µm.

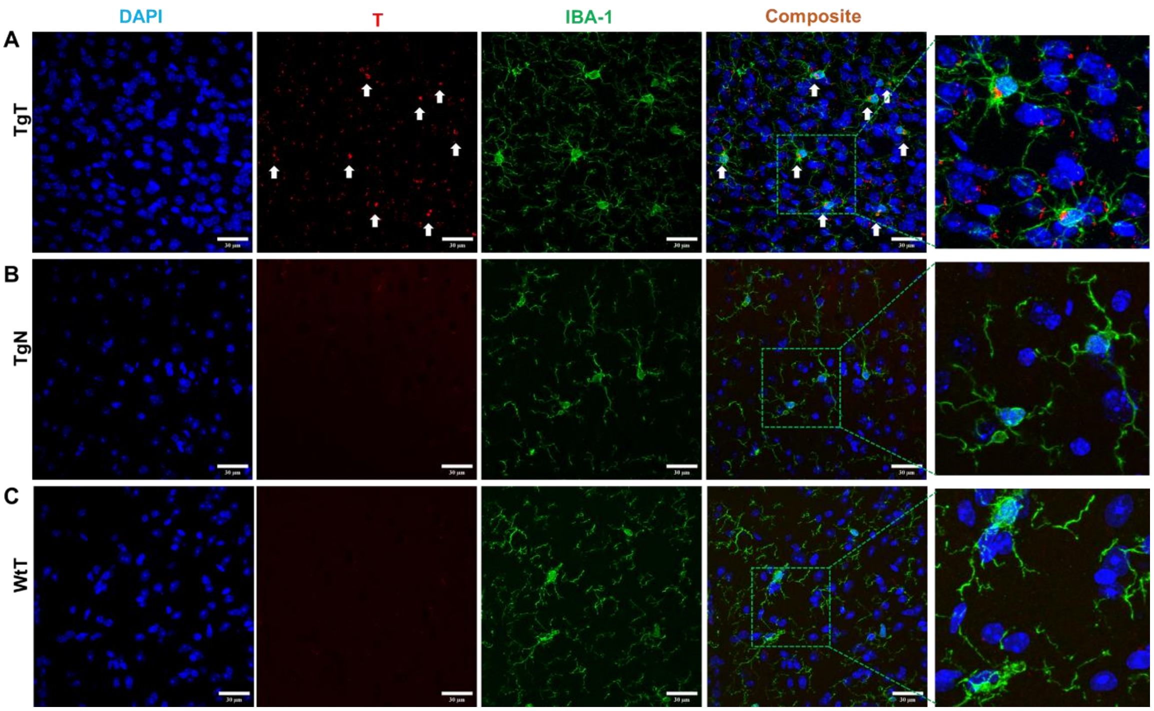


**Figure S11**. Microglia display *in vivo* uptake of nano scavenger species with apparent compact structural formation (white arrows) in the entorhinal cortex as 13 to 15-month-old **TgT**, **TgN** and **WtT** mice all show cell bodies and processes with strong IBA-1 reactivity (**A**) Composite images from imaged **TgT** tissue sections colocalization of NS signal (Rhodamine) in cell bodies of anti-IBA-1 reactive cells; Controls sections, **TgN** and **WtT** (**B** and **C** respectively) show very faint to no signal in the rhodamine channel. Scale bar = 30 µm.

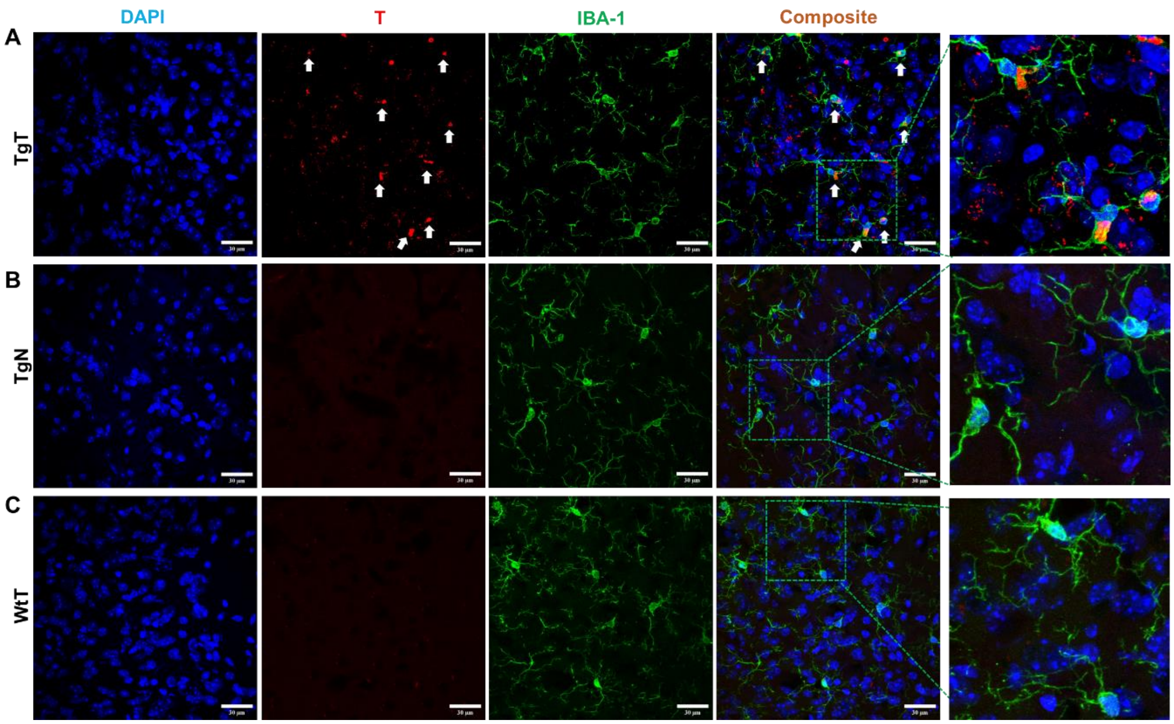


**Figure S12.** Microglia display *in vivo* uptake of nano scavenger species with apparent compact structural formation (white arrows) in the brainstem as 16 to19-month-old **TgT**, **TgN** and **WtT** mice all show cell bodies and processes with strong IBA-1 reactivity (**A**) Composite images from imaged **TgT** tissue sections colocalization of NS signal (Rhodamine) in cell bodies of anti-IBA-1 reactive cells; Control sections, **TgN** and **WtT** (**B** and **C** respectively) show very faint to no signal in the rhodamine channel. Scale bar = 30 µm.

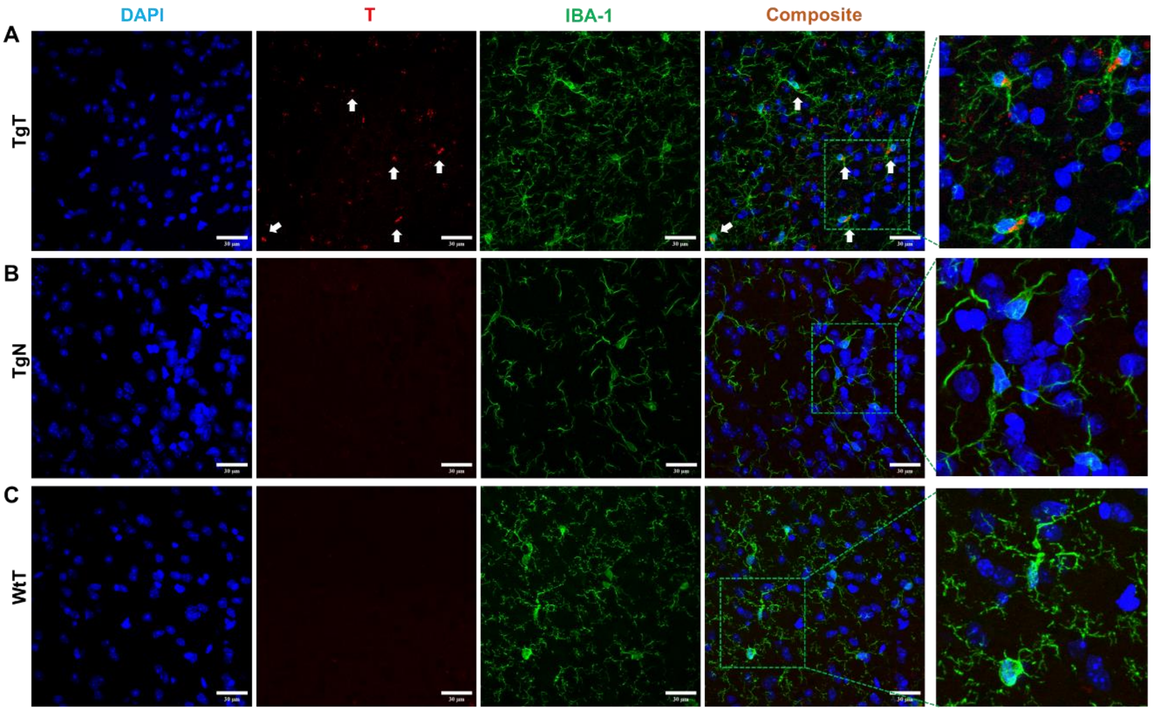


**Figure S13**. Microglia displays *in vivo* uptake of nano scavenger species with apparent compact structural formation (white arrows) in the brainstem as 13 to 15-month-old. **TgT**, **TgN** and **WtT** mice all show cell bodies and processes with strong anti-IBA-1 reactivity (**A**) Composite images from imaged **TgT** tissue sections colocalization of NS signal (rhodamine) in cell bodies of anti-IBA-1 reactive cells; Control sections, **TgN** and **WtT** (**B** and **C** respectively) show very faint to no signal in rhodamine channel. Scale bar = 30 μm.

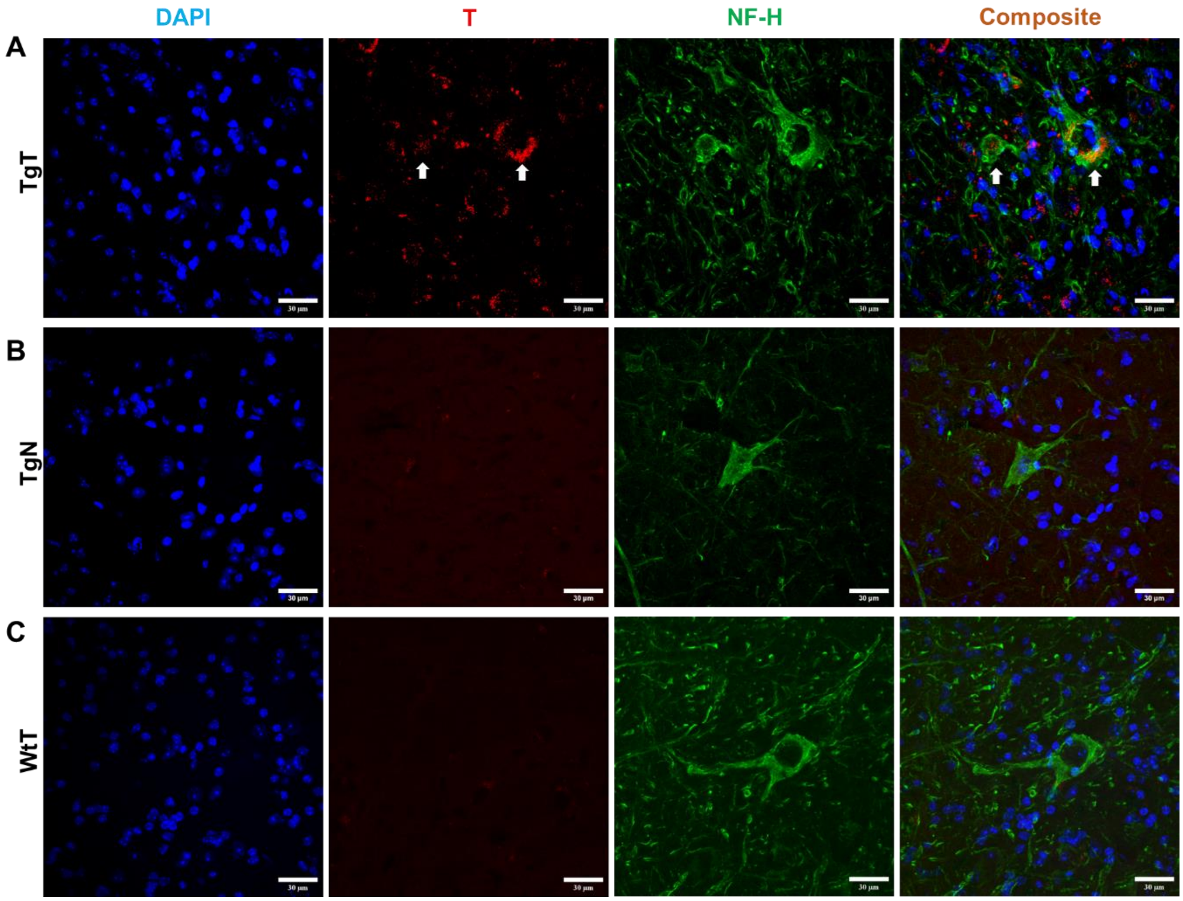


**Figure S14**. Neuronal cell bodies show *in vivo* uptake of nano scavenger species with apparent diffuse structural formation (white arrows). Brainstem sections from 16 to 19-month-old **TgT**, **TgN** and **WtT** mice show cell bodies and processes with strong anti-NF-H reactivity (green fluorescence). (**A**) Composite images of **TgT** sections show that the diffuse rhodamine signal is cytosolic (white arrows); (**B**) and (**C**) **TgN** and **WtT** sections respectively show no signal in the rhodamine channel. Scale bar = 30 µm.

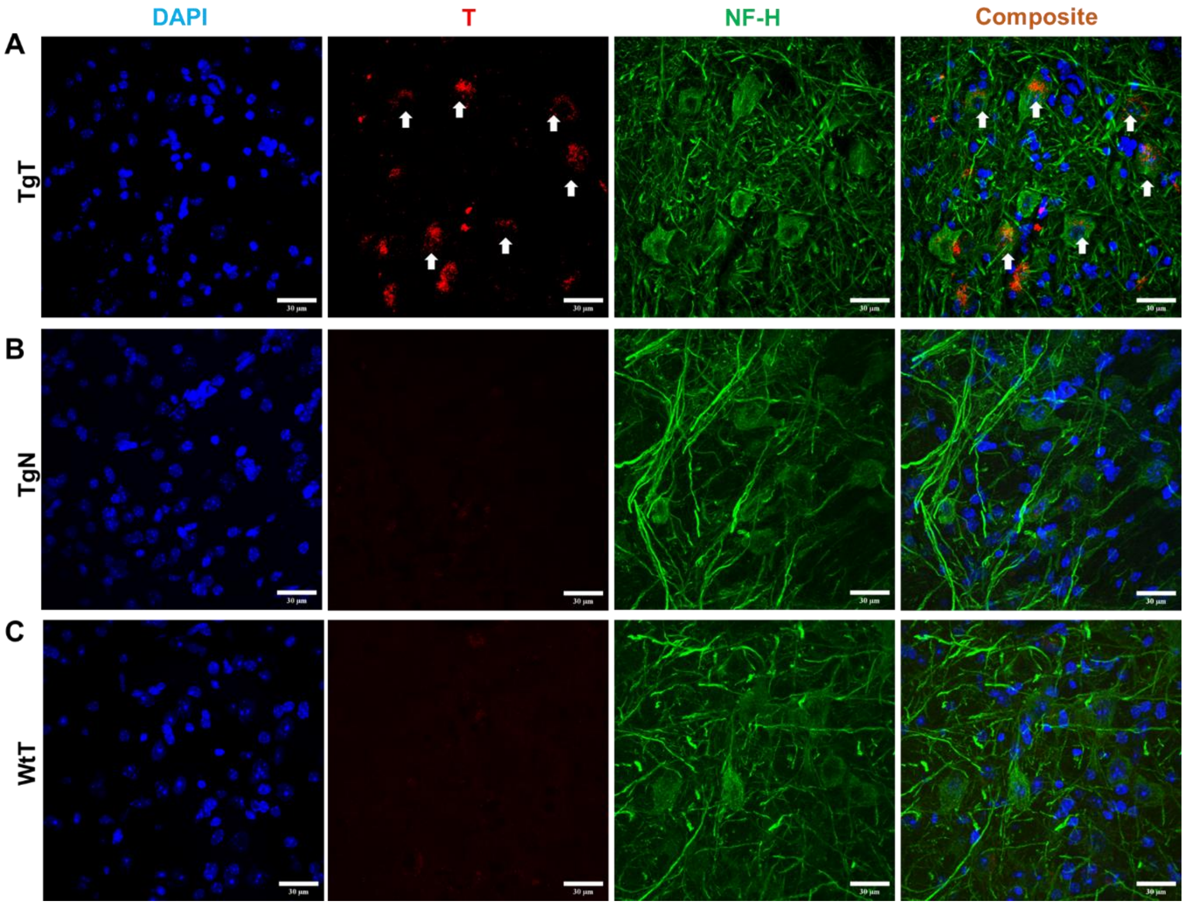


**Figure S15**. Neuronal cell bodies show *in vivo* uptake of nano scavenger species with apparent diffuse structural formation. Brainstem sections from 13 to 15-month-old **TgT**, **TgN** and **WtT** mice show cell bodies and processes with strong anti-NF-H reactivity (green fluorescence). (**A**) Composite images show that the diffuse Rhodamine signal is cytosolic (white arrows). (**B**) **TgN** and (**C**) **WtT** sections show low to no signal in the rhodamine channel. Scale bar = 30 µm.

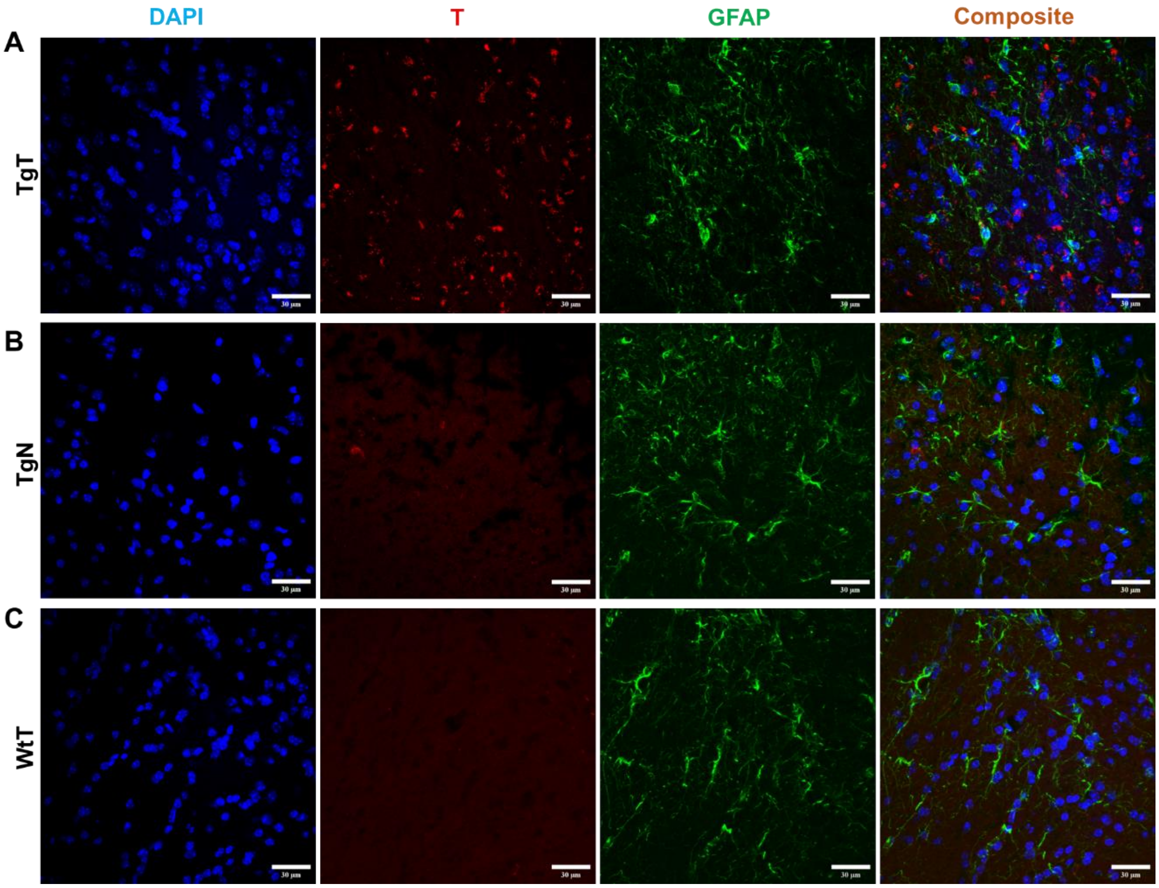


**Figure S16.** Astrocytes do not display convincing evidence of *in vivo* uptake. Olfactory bulb sections from 16 to 19-month-old **TgT**, **TgN** and **WtT** mice show cell bodies and processes with strong anti GFAP reactivity (green fluorescence). (**A**) **TgT** sections show strong NS signal (Rhodamine), but composite images do not show conclusive evidence of internalization of **T** by GFAP reactive cells. (**B**) **TGN** and (**C**) **WtT** sections show no signal in the Rhodamine channel. Scale bar = 30 µm.

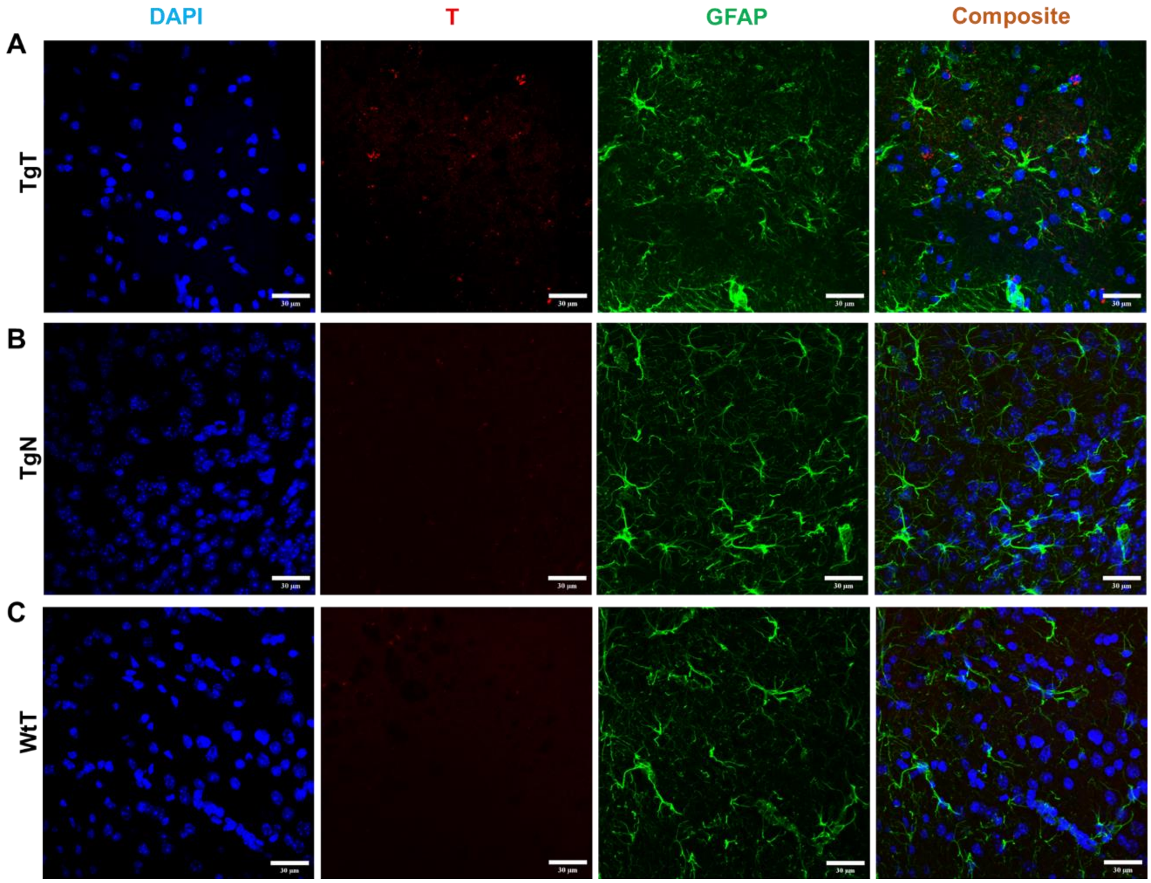


**Figure S17.** Olfactory bulb sections from 13 to 15-month-old **TgT**, **TgN** and **WtT** mice show cell bodies and processes with strong anti-GFAP reactivity. (**A**) **TgT** sections show strong **T** signal (rhodamine) but composite images do not show conclusive evidence of internalization of **T** by GFAP reactive cells. (**B**) **TgN** and (**C**) **WtT** sections show very low to no signal in the Rhodamine channel. Scale bar = 30 µm.

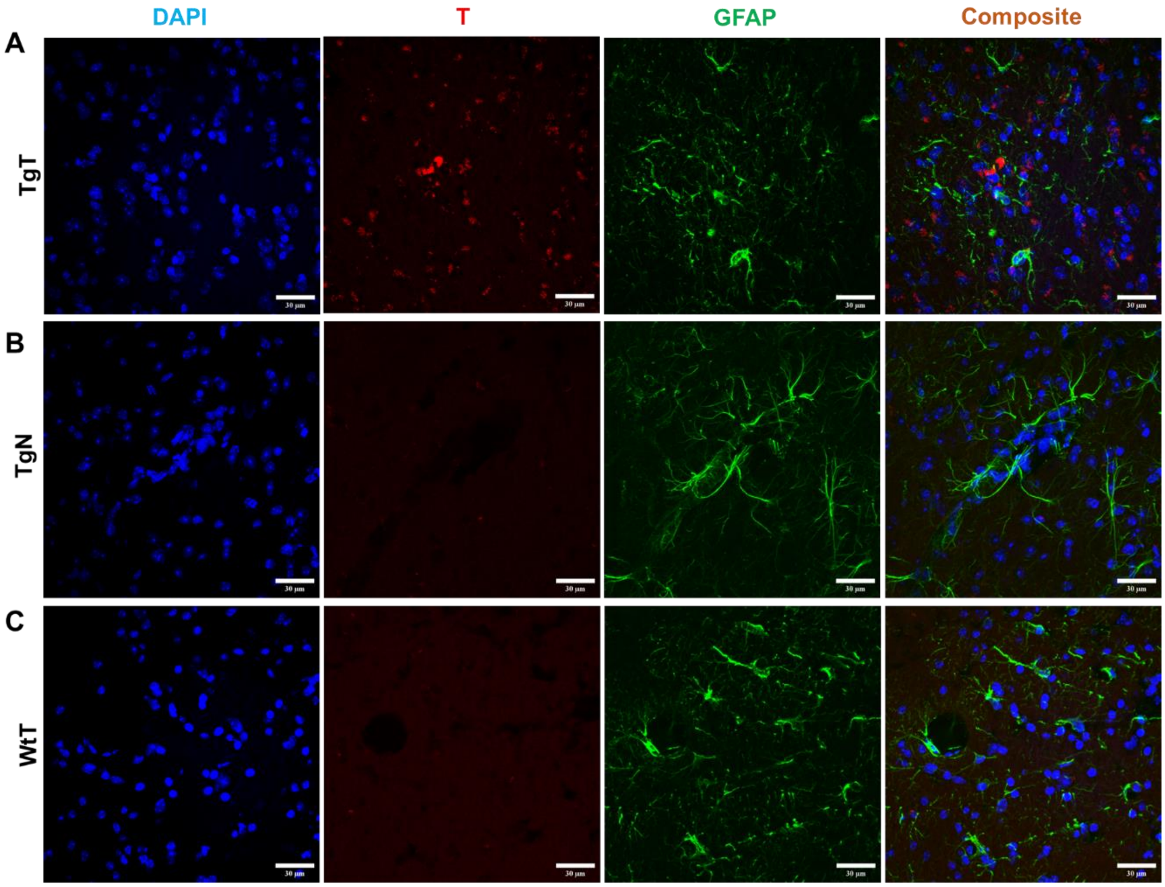


**Figure S18.** Cortical sections from 16 to 19-month-old **TgT**, **TgN,** and **WtT** mice show cell bodies and processes with strong GFAP reactivity (green fluorescence) but no convincing evidence of uptake of **T** *in vivo*. (**A**) **TgT** sections show strong **T** signal (rhodamine), but composite images do not show conclusive evidence of internalization of **T** by anti-GFAP reactive cells. (**B**) **TgN** and (**C**) **WtT** sections show no signal in the rhodamine channel. Scale bar = 30 µm.

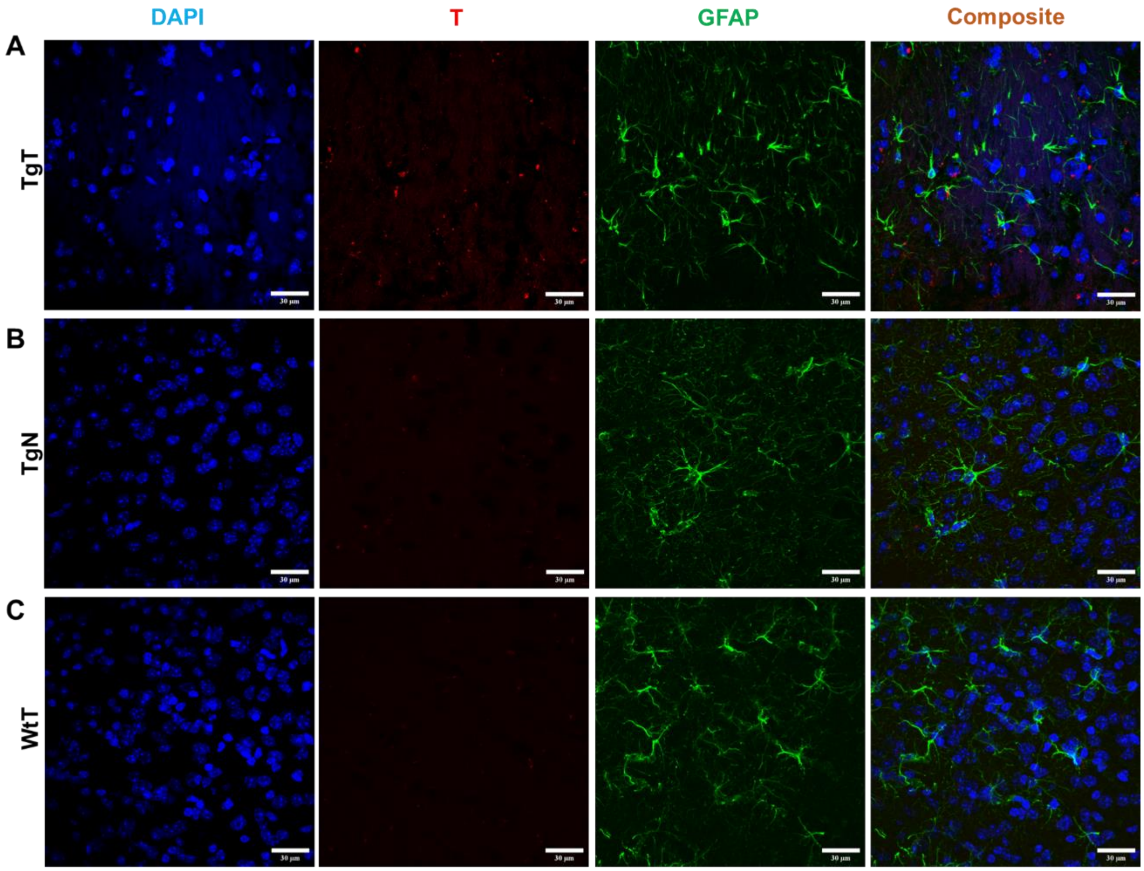


**Figure S19.** Entorhinal cortex sections from 13 to15-month-old **TgT**, **TgN** and **WtT** mice show cell bodies and processes with strong anti-GFAP reactivity (green fluorescence). (**A**) **TgT** sections show strong **T** signal (rhodamine) but composite images do not show conclusive evidence of internalization of **T** by anti-GFAP reactive cells. (**B**) **TgN** and (**C**) **WtT** sections show no signal in the rhodamine channel. Scale bar = 30 µm.

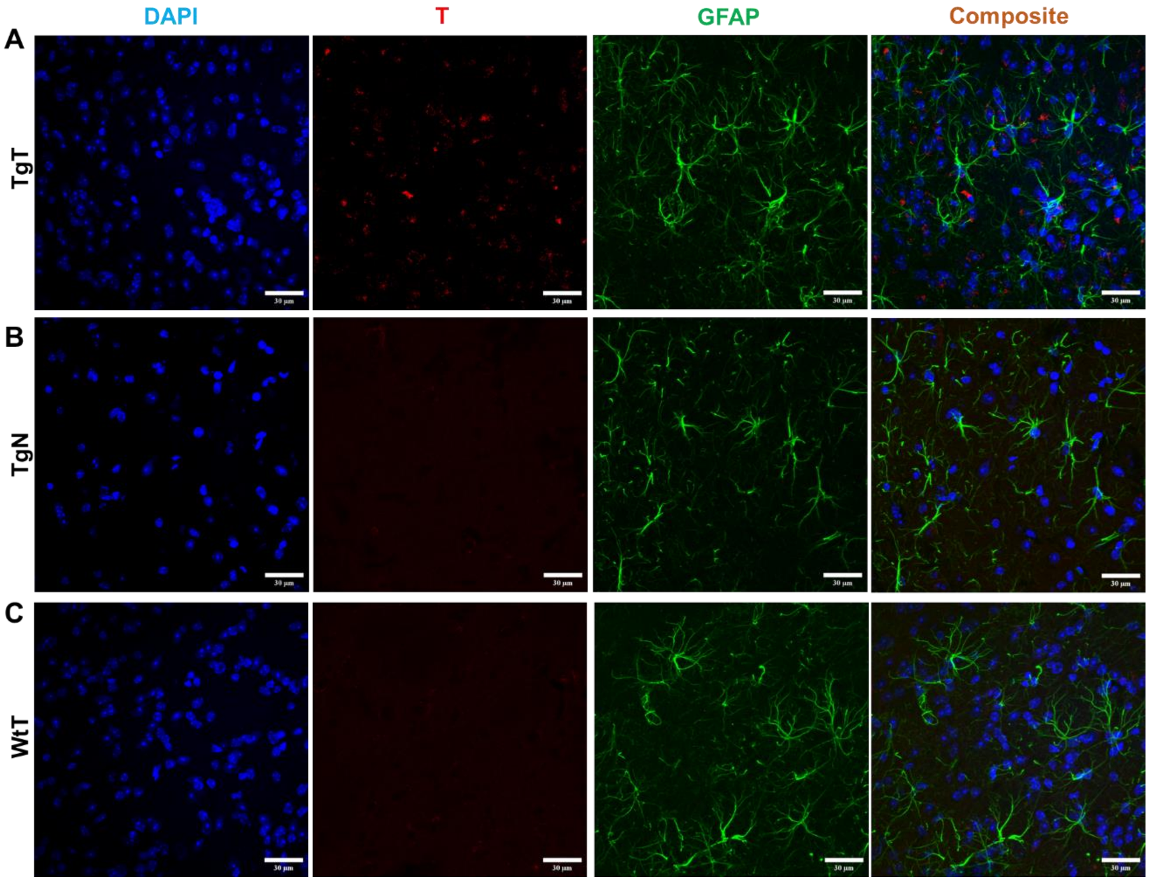


**Figure S20.** Brainstem sections from 16 to 19-month-old **TgT**, **TgN** and **WtT** mice show cell bodies and processes with strong GFAP reactivity (green fluorescence) but no convincing evidence of uptake of **T** *in vivo*. (**A**) **TgT** sections show strong **T** signal (rhodamine), but composite images do not show conclusive evidence of internalization of **T** by GFAP reactive cells. (**B**) **TgN** and (**C**) **WtT** sections show very low to no signal in rhodamine channel. Scale bar = 30 µm.

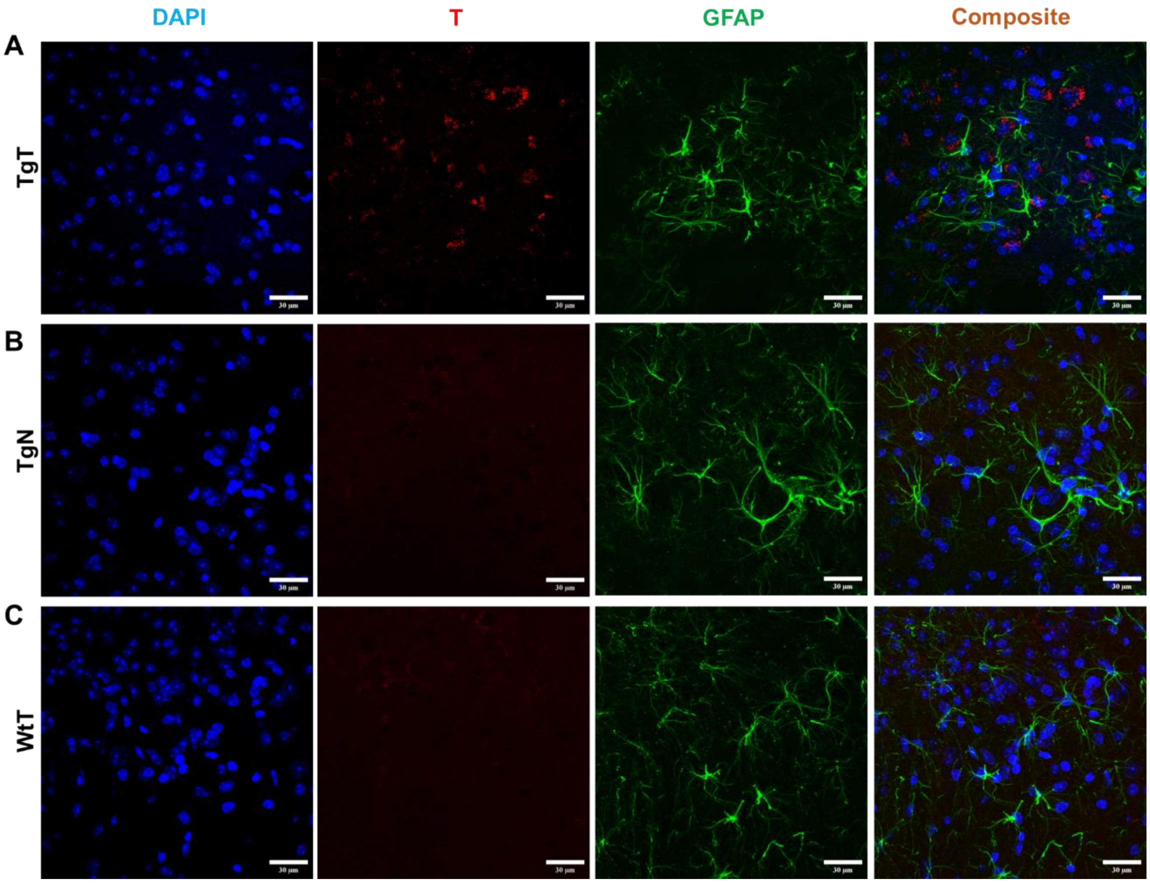


**Figure S21.** Brainstem sections from 13 to 15-month-old **TgT**, **TgN** and **WtT** mice show cell bodies and processes with strong GFAP reactivity (green fluorescence) but no convincing evidence of T uptake *in vivo*. (**A**) **TgT** sections show strong **T** signal (rhodamine), but composite images do not show conclusive evidence of internalization of **T** by GFAP reactive cells; (**B**) **TgN** and (**C**) **WtT** sections show no signal in the rhodamine channel. Scale bar = 30 µm.

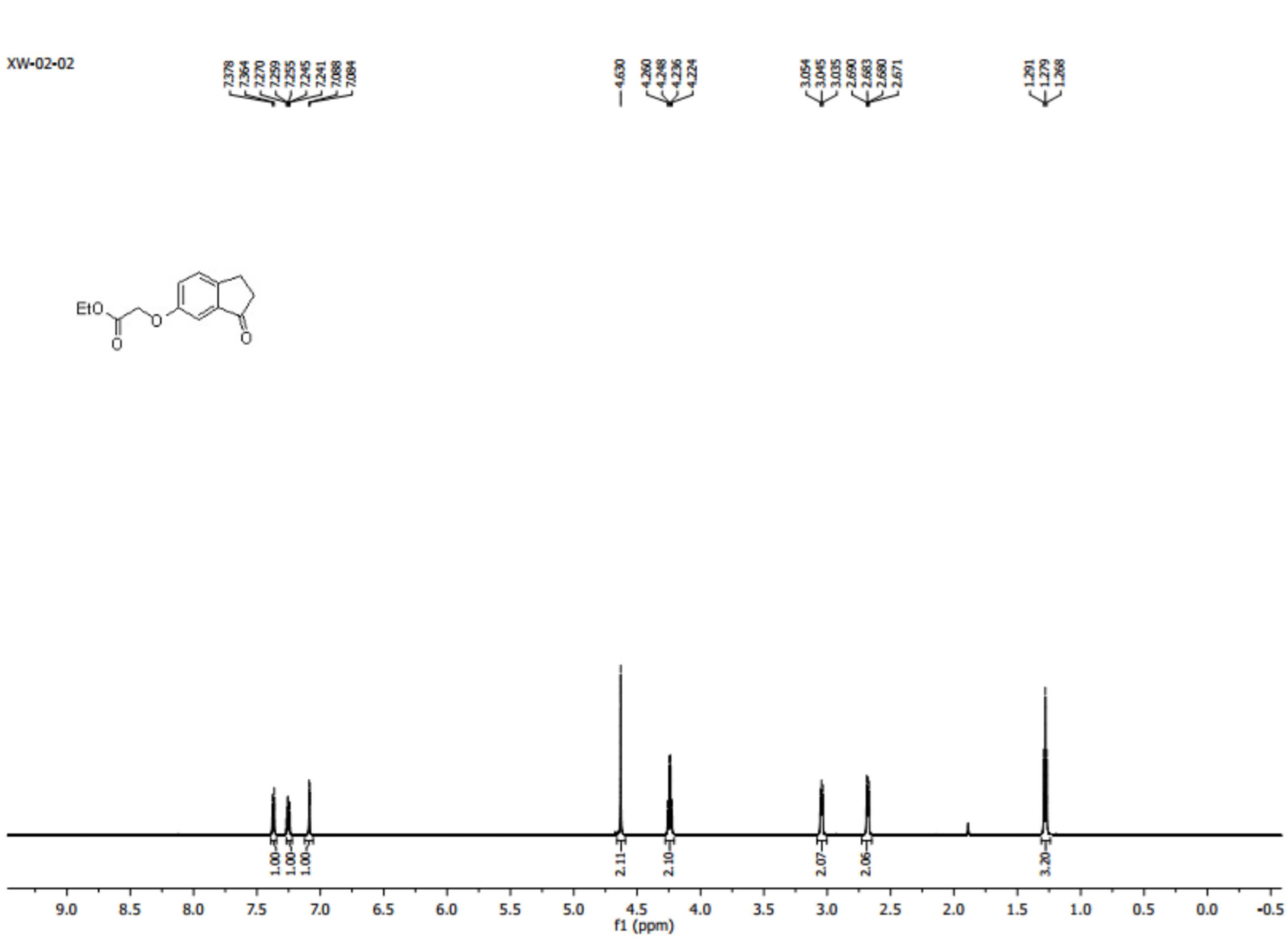


Figure S22. $^1$H NMR Spectrum of Compound 3

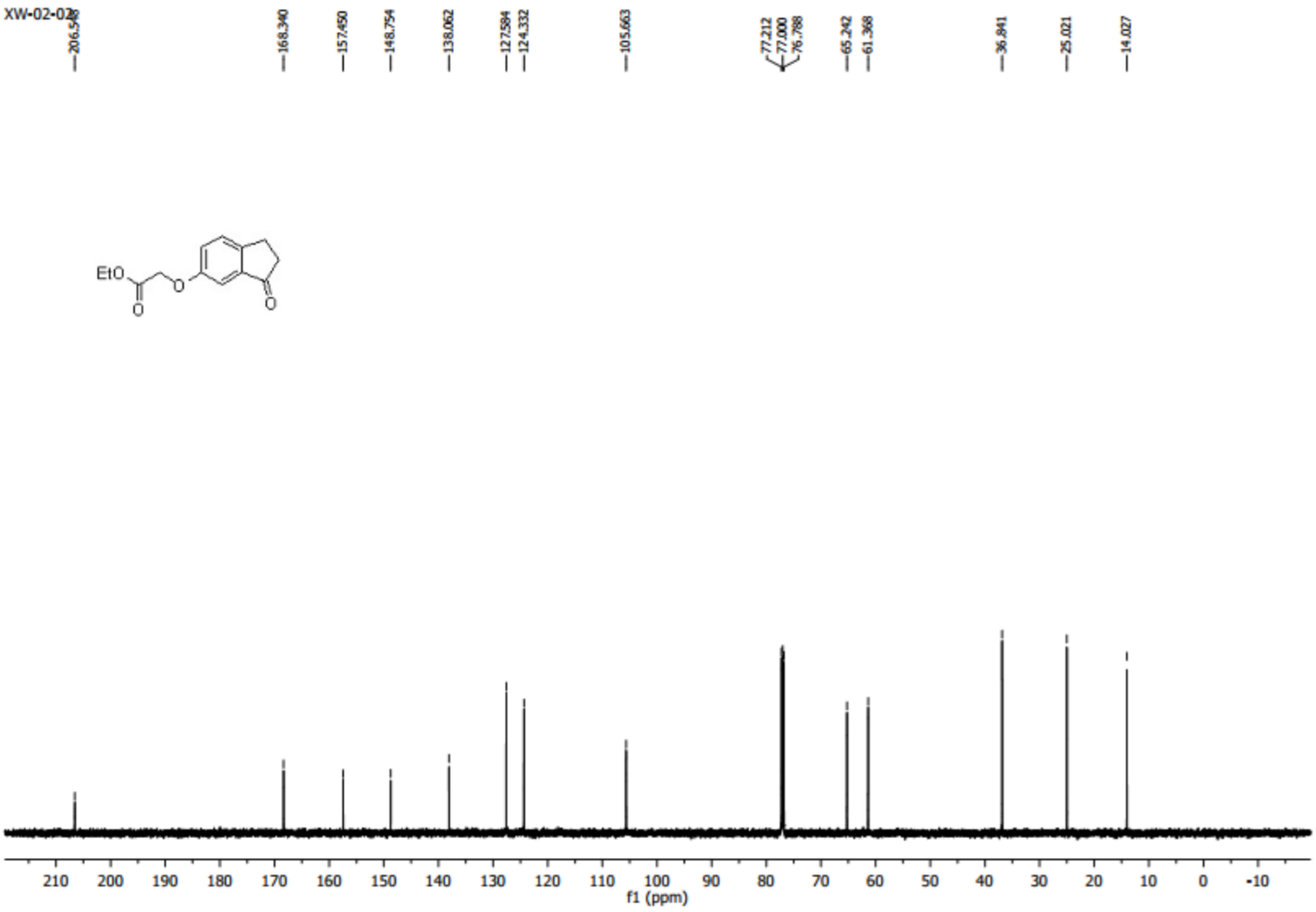


Figure S23. $^{13}C$ NMR Spectrum of Compound 3

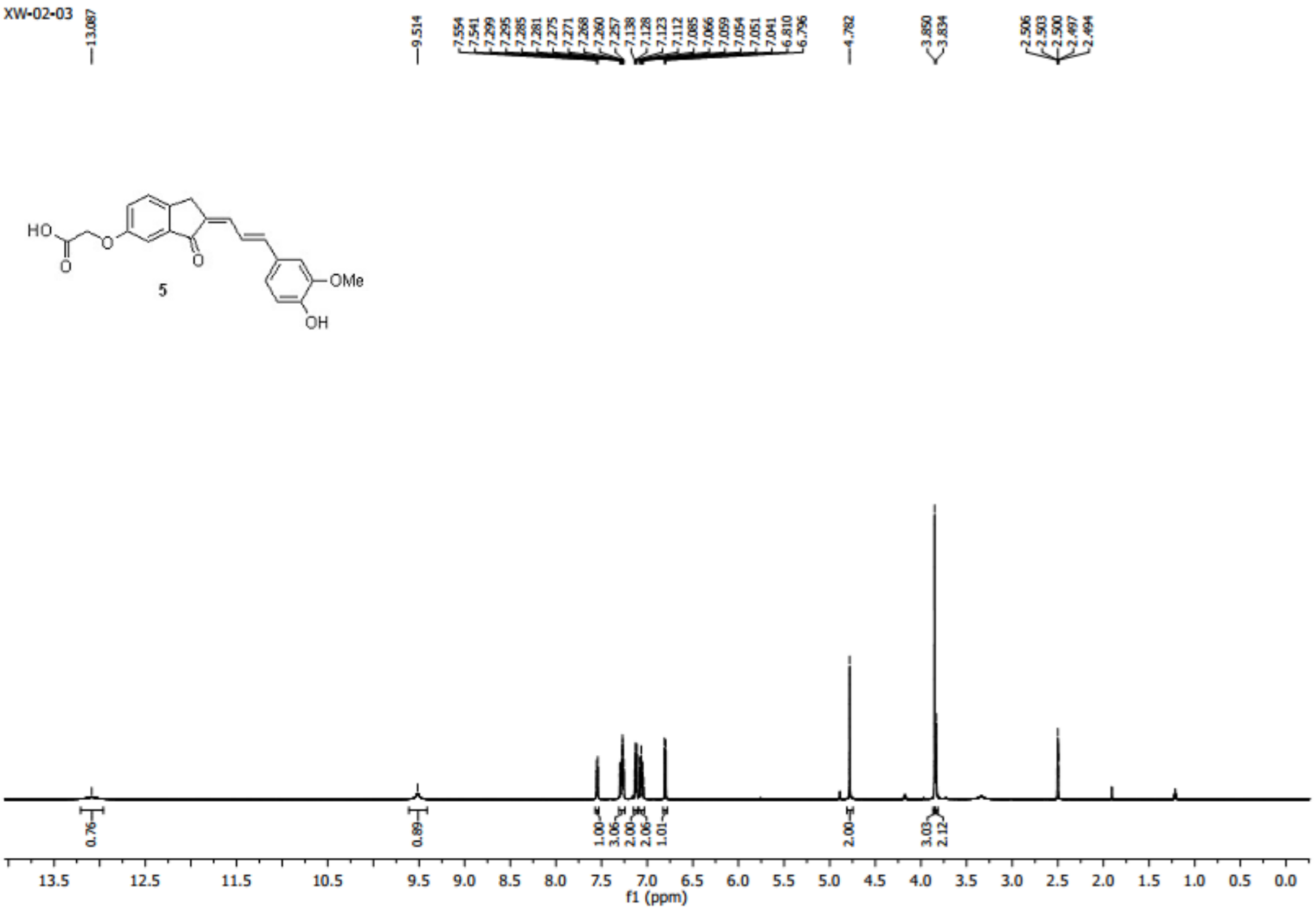


**Figure S24.** $^{1}$H **NMR Spectrum of Compound 5**

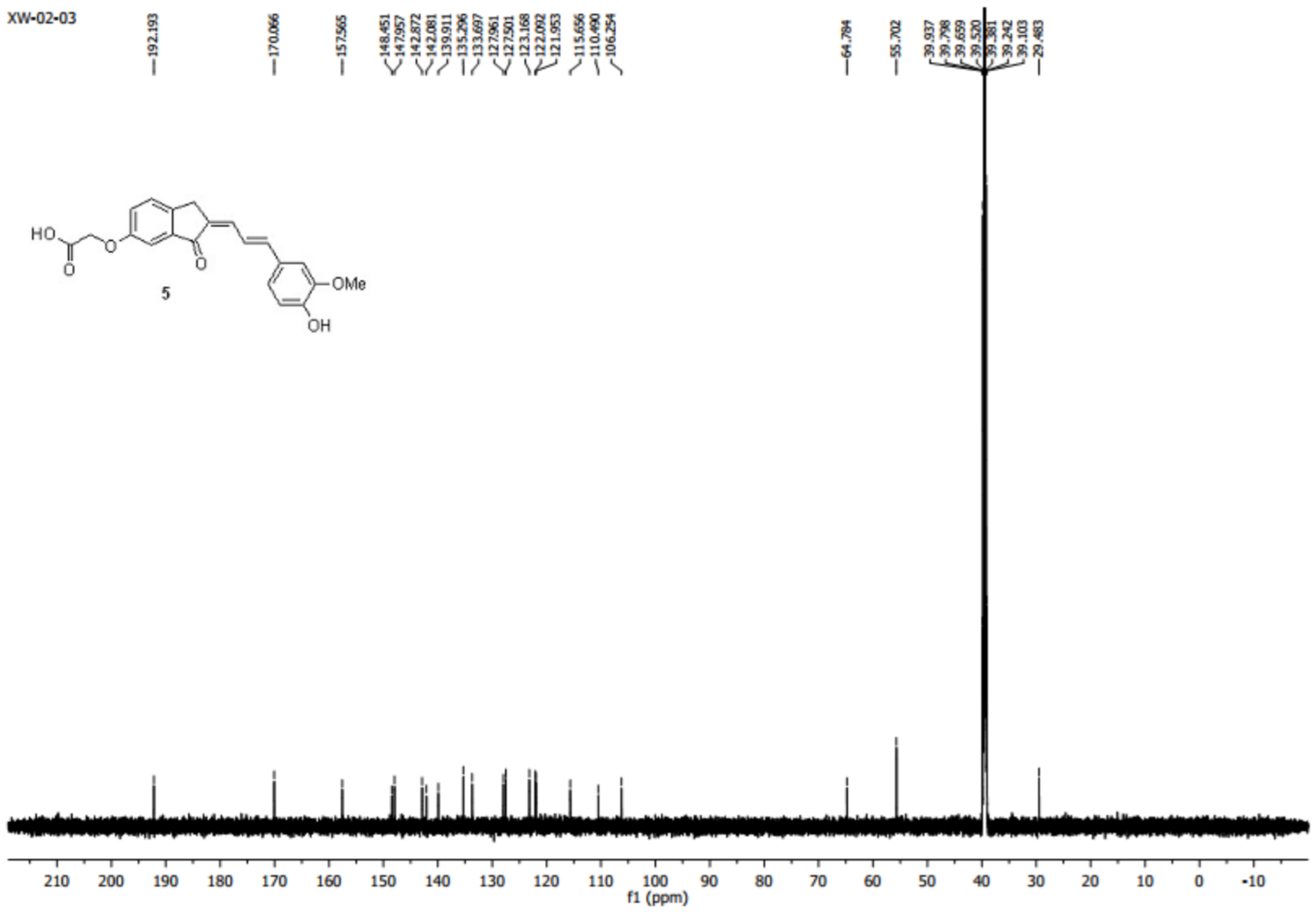


Figure S25. $^{13}C$ NMR Spectrum of Compound 5

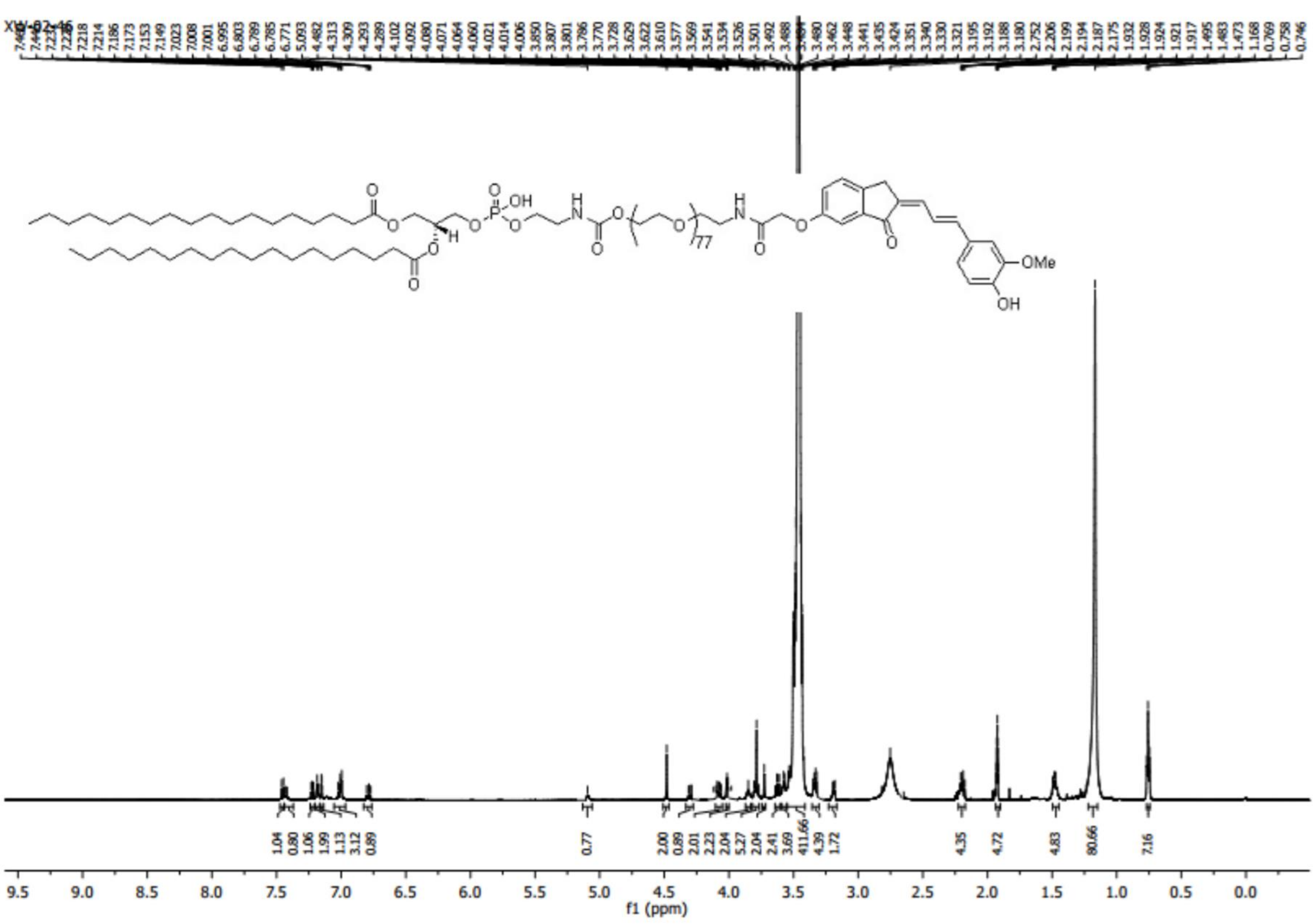


**Figure S26.** $^{1}$H NMR Spectrum of Compound DSPE-PEG(3400)-XW-01-11 Conjugate

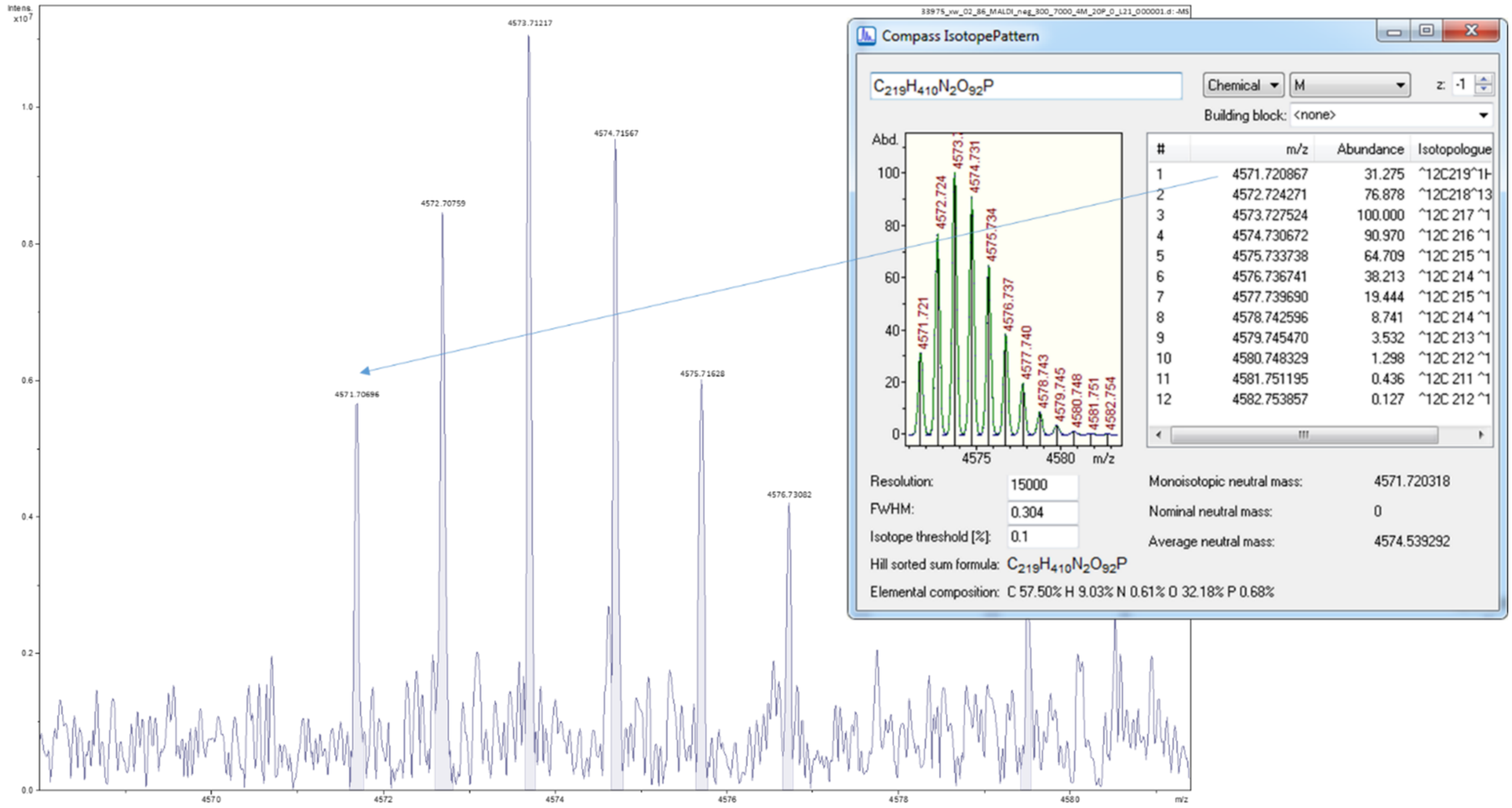


**Figure S27. MALDI Spectrum of Compound DSPE-PEG(3400)-XW-01-11 Conjugate**
